# Perspective: Morphology and ion transport in ion-containing polymers from multiscale modeling and simulations

**DOI:** 10.3389/fchem.2022.981508

**Published:** 2022-08-19

**Authors:** Zhenghao Zhu, Stephen J. Paddison

**Affiliations:** Department of Chemical and Biomolecular Engineering, University of Tennessee, Knoxville, TN, United States

**Keywords:** ion-containing polymers, specific interactions, morphology, ion transport, multiscale modeling, simulations

## Abstract

Ion-containing polymers are soft materials composed of polymeric chains and mobile ions. Over the past several decades they have been the focus of considerable research and development for their use as the electrolyte in energy conversion and storage devices. Recent and significant results obtained from multiscale simulations and modeling for proton exchange membranes (PEMs), anion exchange membranes (AEMs), and polymerized ionic liquids (polyILs) are reviewed. The interplay of morphology and ion transport is emphasized. We discuss the influences of polymer architecture, tethered ionic groups, rigidity of the backbone, solvents, and additives on both morphology and ion transport in terms of specific interactions. Novel design strategies are highlighted including precisely controlling molecular conformations to design highly ordered morphologies; tuning the solvation structure of hydronium or hydroxide ions in hydrated ion exchange membranes; turning negative ion-ion correlations to positive correlations to improve ionic conductivity in polyILs; and balancing the strength of noncovalent interactions. The design of single-ion conductors, well-defined supramolecular architectures with enhanced one-dimensional ion transport, and the understanding of the hierarchy of the specific interactions continue as challenges but promising goals for future research.

## Introduction

Ion-containing polymers are an emerging class of materials including ionic polymers with counter ions and nonionic/neutral polymers doping with ionic material of low molecular weight, combining the advantages of a polymer matrix with mobile ions. They exhibit versatile and attractive properties and have attracted considerable interest as solid electrolytes for a variety of applications. They selectively conduct ions but not electrons. They are broadly used in energy conversion and storage devices, electrodialysis for water treatment, and solar-driven desalination. Next-generation ion-conducting polymeric materials are being developed for fuel cells, lithium batteries, electrolyzers, and other electrochemical devices. In fuel cells, a fuel such as: hydrogen, methane, or methanol is efficiently converted into electrical energy with the aid of catalysts with the typical byproduct being only water. This contrasts with solid-state Li-ion battery electrolytes where there must be no water present because of the reactive materials used in the electrodes. Thus, the presence of water is a crucial parameter in distinguishing the electrolytes suitable for fuel cells from those employed in some batteries. Differences in these materials including the coupled dynamics of the mobile ions and the chains naturally lead to different ion transport mechanisms and consequently design principles.

Over the past several decades, three main types of polymeric electrolytes: proton exchange membranes (PEMs), anion exchange membranes (AEMs), and polymerized ionic liquids (polyILs) have gained fundamental understanding and hence improvement from experimental work and theoretical studies (Kusoglu and Weber, 2017; Elabd, 2019; Wang and Park, 2020; Ma and Olvera de la Cruz, 2021; Shen et al., 2021). PEMs were commercialized to some extent because of their high proton conductivity and first use in the Gemini space program in the early 1960s (Kusoglu and Weber, 2017). However, the requirement of expensive noble metal catalysts impedes the application in the real world and provokes additional research into alternative PEMs. AEMs have also attracted significant attention as an alternative to PEMs in fuel cells due to the use of non-noble transition metals as electrocatalysts under the high OH¯ environment even though their conductivity is typically much lower (Dekel, 2018; Elabd, 2019). Thus, substantial effort has been expended to investigate the mechanisms underlying ion transport and improve the performance of AEMs. In view of the use of polymer electrolytes in batteries, polyILs, in which each repeating unit may be charged as opposed to ionomers that contain a relatively small percentage of charged units, are promising alternatives to organic liquid-based electrolytes that are toxic, flammable, and have a limited electrochemical stability window, due to their generally good mechanical stability, flexibility, improved safety, and durability. However, it remains challenging to enhance the ion conductivity of polyILs to a sufficient degree for electrochemical applications due to the sluggish local dynamics of the polymer chains, low dielectric constant, and small fraction of mobile ions in this class of materials (Bocharova and Sokolov, 2020; Son and Wang, 2020).

The performance of ion-containing materials is influenced by a variety of tunable factors, including: the functionalized end-groups; composition of block copolymers; polarity and rigidity of polymer backbones; molecular weight; conformational asymmetry; etc. Such factors provide an opportunity to tailor the structural and dynamical properties of the materials, although they bring challenges to identifying broadly applicable design rules. We hypothesize that the origins of how these factors affect the molecular packing and the dynamics of ions are mainly from the relatively weak specific interactions in comparison to covalent bonds as they play a key role in block copolymers (Wang and Park, 2020). This provokes several important but as of yet unanswered questions: How do these specific interactions affect the properties of an ion-containing polymer? Do the specific interactions have synergistic or uncooperative effects? Which interactions are the most dominant or important? Hence, the development of more advanced ion-containing polymers depends on a better understanding of specific interactions of polymer/polymer, polymer/solvent, acidic or basic group/solvent, and fixed-/counter-ions. These interactions including hydrogen bonding, ion-dipole interactions, Coulombic, and acid-base interactions may govern molecular arrangements, selectivity of ion uptake and transport, and ion transference.

In determining the role of the specific interactions on the performance of a material, experimental techniques have been employed to study the structural and dynamical properties of ion-containing polymers at various length and time scales. For example, small angle x-ray scattering (SAXS), wide angle x-ray scattering (WAXS), small angle neutron scattering (SANS) have been widely applied to deduce hydrated morphology (Gebel and Diat, 2005), and quasi-elastic neutron scattering (QENS) (Perrin et al., 2007; Melchior and Jalarvo, 2019) and pulsed field gradient NMR (PFG-NMR) (Münchinger and Kreuer, 2019) to measure the self-diffusion coefficient of the ions and water molecules, and broadband dielectric spectroscopy (BDS) (Giffin et al., 2013) to compute the ion conductivity. Other techniques have been brought to bear including infrared (IR) spectroscopy (Zimudzi and Hickner, 2016), Raman spectroscopy (Meek et al., 2020), atomic force microscopy (AFM) (Economou et al., 2015), and transmission electron microscopy (TEM) (Allen et al., 2015; Zhang et al., 2021a), with which one can obtain a description of the molecular interactions, direct images of the surface and 3-D morphology.

**FIGURE 1:**
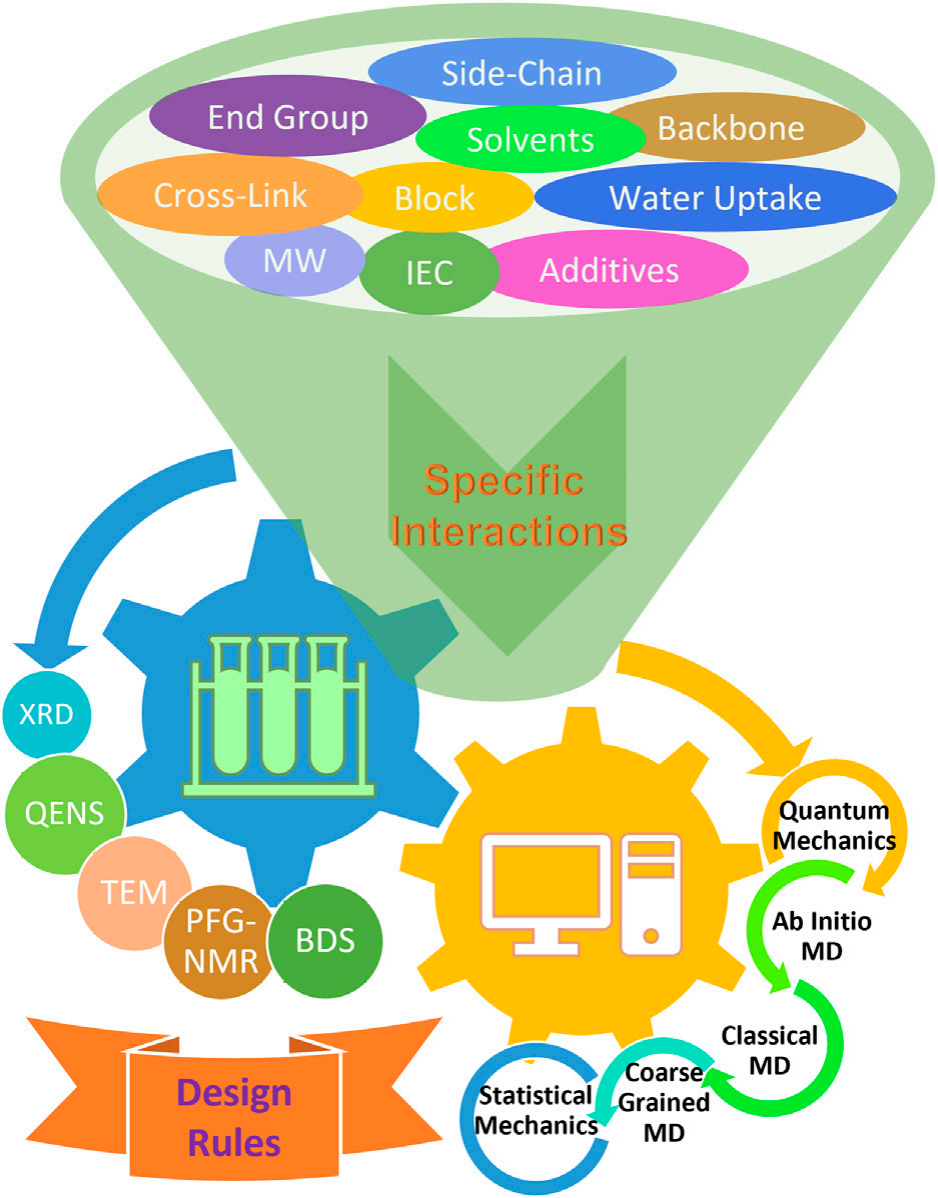
Schematic summary of experimental and theoretical approaches used to study the effects of various chemical factors in pursuit of rational design strategies. Although the understanding of non-bonded specific interactions is in its infancy, it is recognized that they may play a key role in the dynamic properties within the local environment and structural formation due to long-range effects. Many techniques have been widely utilized to study the effects of various chemical and structural factors with the aid of theoretical modeling and simulations in an effort to discover general design rules for advanced ion-containing polymeric materials.

Although advances in technology provide powerful and robust tools to elucidate important information on these materials, modeling and simulations are clearly necessary to complement experiment findings and guide further experimental directions with the purpose of enhancing our understanding of morphology formation and ion transport mechanisms as well as the development of improved materials for electrochemical applications. Given the complex morphological and dynamic behavior of ion-containing polymers, multiscale theoretical approaches are required to describe characteristics over a wide range of temporal and spatial scales. Quantum mechanical calculations and *ab initio* molecular dynamics (AIMD) simulations are regarded as highly accurate methods to gain information on the static and dynamic properties allowing for both the electronic and nuclear degrees of freedom, which are critical when studying proton/hydroxide ion diffusion since the associated proton transfer involves the breaking and forming of covalent bonds. However, the use of AIMD is limited to access small time and length scales. To expand the systems up to the size of a hydrophilic pore or domain, multistate empirical valence bond (MS-EVB) (Chen et al., 2016) and reactive ReaxFF (Zhang and Van Duin, 2015) methodologies may be employed to study both vehicular diffusion and structural diffusion in hydrated ion exchange membranes, but it is still challenging to capture the solvation structures of proton/hydroxide ions and to transfer the force fields to different chemical structures. When dealing with nonreactive systems such as polyILs, classical molecular dynamics (MD) simulations are suitable in investigating the structure and ion transport at time scales up to microseconds. The deficiency is the difficulty in allowing for polarization effects, which is resolved by either developing a computationally expensive polarizable force field (Bedrov et al., 2019) or simply scaling the full charges by some fraction. The latter was recently proven to be capable of capturing polarizability effects on both structural and dynamic properties of polyILs based on the comparison of the results obtained from the scaled partial charge force field and the Drude oscillator polarizable force field (Zhang et al., 2022). Although classical MD simulations are capable of predicting transport phenomena and structures within a small region, due to the limitations of time (<1 μs ) and length (<10 nm) scales, coarse-grained MD and dissipative particle dynamics (DPD) simulations are widely utilized in modeling phase separations within membranes that typically require large length scale of 10–200 nm (Zhu et al., 2022). To capitalize on both experimental and theoretical strategies, a tight feedback loop of modeling and simulations with a broad spectrum of experimental techniques will serve as a protocol for the rational design of functional materials with optimal properties (Figure 1).

This Perspective describes recent and significant results regarding the specific interactions, morphology, and ion transport within ion-containing polymers. Emphasis is put on the interplay of morphology and ion transport. Specifically, we discuss the influences of polymer architecture, choice of the tethered ionic group, the rigidity of the polymer backbone, presence of a solvent, and additives on the morphology and ion transport. Finally, we highlight the challenges and prospects in advancing the performance of ion-containing materials, including the effects of specific interactions, the design of single-ion conductors, and well-defined supramolecular architectures with one dimensional enhanced ion transport channels.

## Specific interactions

Through decades of research into ion-containing polymers, important fundamental understanding of structure-property relationships has been elucidated. However, challenges remain, including addressing the following issues: how to retain water at high temperatures to improve water-mediated ion transport; how to synergistically achieve mechanical stability and fast ion transport; how to facilitate decoupling of the ion transport from segmental dynamics or viscosity; and how to organize tethered ionic groups to facilitate efficient transport of ions. To resolve these issues, the specific interactions have been thought to play a crucial role in governing the microstructure and dynamics of the ions in such materials, which is a promising perspective for future research and development.

**FIGURE 2:**
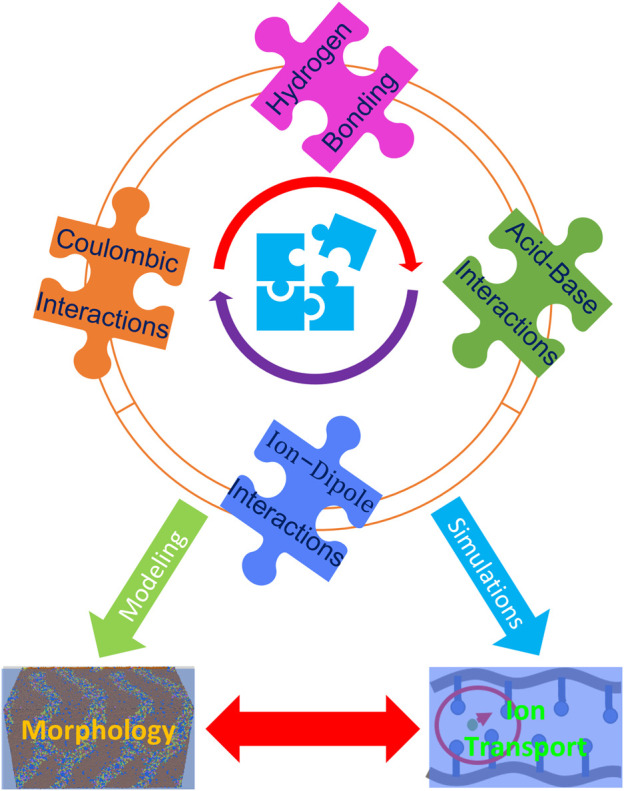
Schematic illustration of the pieces of “the puzzle” including specific interactions that need to be resolved to control morphology and dynamical properties. Coulombic, hydrogen bonding, acid-base, and ion-dipole interactions all play a crucial role in the performance of ion-containing polymers. Tuning these interactions with innovative strategies, though challenging, is an important direction in the design of high-performance materials.

These interactions include hydrogen bonding, acid-base interactions, Coulombic interactions, ionic interactions, and π-π stacking (which may govern molecular arrangements). In view of the need to devise rational design rules, a hierarchy of intermolecular interactions should be fundamentally investigated through theoretical computations and simulations tightly coupled with advanced experimental strategies. This information should lead to insight into tailoring properties by balancing the specific interactions between fragments or molecules. It is a challenging but promising target to resolve this “puzzle” of the various specific interactions within ion-containing polymers in order to guide the design of high-performance materials (Figure 2).

Hydrogen bonding is the most well known and thoroughly investigated interaction and has led to an adequate understanding of the nature of bonding and functionality. It plays a crucial role in determining the water uptake in ion exchange polymer systems and the miscibility of polymer chains. The combination of *in situ* FTIR spectroscopy, molecular dynamics simulations, and statistical thermodynamics methods has been employed to investigate the effects of these interactions on the local structures of a hydrated poly(ether imide)(PEI) system (De Nicola et al., 2017). The authors (De Nicola et al., 2017) concluded that water molecules tended to form “bridges” connecting two successive intrachain carbonyl groups of PEI through hydrogen bonding and water-water interactions led to a “second shell” hydration layer.

The impact of acid-base interactions between sulfonic acid groups in a PEM and incorporated basic groups on the physicochemical properties of a membrane was investigated by synthesizing novel multi-block PEMs with three different basic groups (i.e. benzoxazolium, benzotriazolium, and propylamino groups) (Sui et al., 2019). The results reveal that a PEM with benzotriazolium (with an intermediate p*K*_a_) had the highest proton conductivity although the excessive water absorption and the membrane swelling were suppressed in all three membranes. It was reasoned that the moderate basicity forms a “protonation-deprotonation” loop between the hydrophobic and hydrophilic moieties reducing the proton transfer barrier. Strong basicity was thought to neutralize the membrane acidity thereby lowering the concentration of dissociated (i.e., mobile) protons.

Ionic interactions between fixed ionic groups and mobile ions have an influence on the ionomer’s local structure, hydration, ion transport within the ionic domain, and even the polymer chain segmental motion/dynamics. This type of interaction is strongly dependent on the hydration level since their effect is shielded and diminished as the water content is increased. The micromorphology of block copolymers was tailored by casting with various counter-cations (i.e. H^+^, Li^+^, K^+^, Cs^+^, TEA^+^, and Ca^2+^), which acted as either a block compatibilizer or a block separator by tuning the ionic interactions (Nguyen et al., 2020). The choice of counter-cations can vary the phase-separated morphologies from disordered to highly ordered, and thereby the conductivity of the membrane. Shi et al. (Shi et al., 2020) studied the role of ionic interactions in the deformation and fracture behavior in model perfluorosulfonic acid (PFSA) membranes. They determined that the ionic crosslinks formed between the cations and the sulfonate anions of the polymer enhance the alignment and stretching of the polymer matrix and the effect decreases with the cation radius. Another theoretical study (Ghosh and Chakrabarti, 2016) showed that small sodium ions favorably reside around the sulfonate groups due to electrostatic interactions while bulky tetramethylammonium ions tend to stay near the hydrophobic backbones because of strong hydrophobic interactions in Nafion membranes.

Recently, these specific interactions between fixed-ions and counter-ions were also confirmed in Nafion, Aquivion, sulfonated polystyrene, sulfonated polyether ketone, sulfonated poly(phenylene sulfone), poly(acrylic acid) and ammonium functionalized poly(phenylene oxide) ionomers (Münchinger and Kreuer, 2019). The experimental observation of the different degrees of localizing Li^+^ and Cs^+^ by the dissolved ionomers suggests that the ion partitioning seems to be controlled by the acidity of the fixed ionic groups but not electrostatics. Explicitly, the ratio of [Li^+^]/[Cs^+^] decreases with increasing acidity, which ascribes to stronger stabilization of Cs^+^ in comparison to Li^+^. The diffusivity of Cs^+^ decreases as the acidity of the fixed ionic group increases, while the trend is inversed for Li^+^ before the onset of counter-ion binding, similar to the behavior of H^+^. It was concluded that the strongest specific interaction exists between the most acidic fixed functional groups and cations with the highest polarizability and lowest electronegativity.

In summary, there exists several interactions between the various molecules and ions in these materials. It is worth noting that π-π stacking interactions play a crucial role in promoting micro- or nano-phase separation and stabilizing porous structures. However, it is still at nascent stage especially in ion-containing materials since these interactions require delicate description of π systems allowing for electron density. It is crucial to elucidate how the interactions affect the morphology and dynamics, which are discussed in the following sections. To develop the desired properties, we have to balance the interactions *via* various approaches. For instance, the addition of nanoparticles into polymeric materials can reduce the hydrogen bonding between the cations and anions, improving the dissociation of ions by balancing the interaction of nanoparticles with cations and anions, which has been proven by the FTIR and the Raman spectroscopy (Pundir et al., 2018).

## Morphology

Understanding nanophase separation (i.e., characteristics of the hydrophobic domain in comparison to the ion conducting phase) in ion-containing polymeric systems is fundamentally important in the rational design of highly conducting electrolytes with mechanically and chemically stable integrity under diverse operating conditions. Generally, these polymers possess hydrophobic and hydrophilic components, which provoke phase separation of ionic domains from the polymeric matrix when exposed to polar solvents. Morphological differences are governed by various factors, such as the dissimilarity between pendant acidic or basic groups and the backbone, equivalent weight, side-chain chemistry, degree of hydration, *etc*. The underlying fundamentals of how these factors affect morphology are of significant interest.

### Hydrated PEM morphology

Phase separation upon hydration enhances the formation of pore networks that provide pathways for the movement of water and ions. The hydrophobic polymer matrix provides dimensional strength. The connectivity of the pore network is key to promoting long-range ion transport, which ultimately determines conductivity. An increase in hydration may improve phase separation by establishing percolating pore networks, but high-water content may also cause the swelling of membranes resulting in the reduction of mechanical stability and constraint in the operating temperature below the boiling point of water. It is recognized that an increase in temperature enhances reaction kinetics and reduces the poisoning of the catalysts (Kusoglu and Weber, 2017). Therefore, it is desired, on one side, to form well-connected channels in membranes at low relative humidity; on the other side, to retain water in the ionic domains of membranes at higher operating temperatures. Nevertheless, there are various factors including polymeric architecture, degree of functionalization, pendant ionic groups, counterions, and hydration level, which play a complex role in the hydrated morphology of ion-containing membranes. It is impractical to conduct a case-by-case study to elucidate the interplay of the multitude of these factors. Moreover, the model-dependent interpretation of scattering data cannot provide precise geometries of the multiscale domains, which has been extensively discussed for perfluorosulfonic acid ionomers (Kusoglu and Weber, 2017).

Given these challenges of unambiguously determining the morphology, theoretical approaches have been resorted to providing insight into the quantification of morphology for ion-containing membranes. Coarse grained MD and DPD simulations are widely utilized in modeling phase separations within membranes that typically requires long-time relaxation and a large length scale of 10–200 nm (Dorenbos, 2017a; Dorenbos, 2017b; Sepehr et al., 2017; Liu et al., 2018a; Dong et al., 2018b; Liu et al., 2018b; Wang R. et al., 2019; Clark et al., 2019; Dorenbos, 2019; Lee, 2019; Luo and Paddison, 2019; Zhu et al., 2019; Chen C. et al., 2020; Luo et al., 2020a; Dorenbos, 2020; Lee, 2020; Sevinis Ozbulut et al., 2020; Zhu et al., 2022). To further quantify the morphology including the size, shape, and connectivity of the ionic domains, which cannot be extracted from the peaks obtained by scattering methods, cluster analysis including distance-based and density-based algorithms is a powerful tool to provide a plethora of information on water domain size, shape, and connectivity from the trajectory of a MD or DPD simulation, which will be described in this section. The latter is capable of characterizing the local regions of high density and isolating clusters that have some overlap (Clark et al., 2019; Sorte et al., 2019).

**FIGURE 3:**
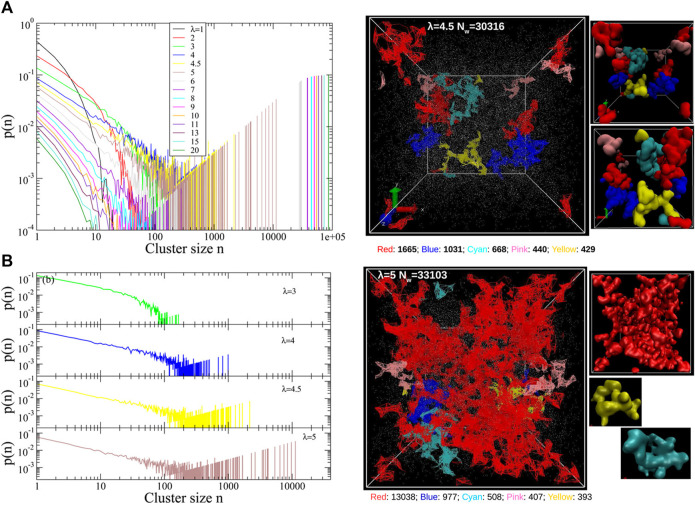
**(A)** Quantitative cluster size distribution from a distance-based cluster analysis algorithm for various hydration levels. **(B)** Cluster size distributions underlining the percolation threshold at λ = 5. Snapshots of water clusters for λ = 4.5 (no percolating clusters) and λ = 5 (percolating clusters) color-coded by the unique cluster IDs on the right panel. Reprinted and adapted with permission from ref. (Liu et al., 2018a). Copyright 2018 American Chemical Society.

Nafion is the prototypical perfluorosulfonic acid (PFSA) ionomer and has been subject to extensive investigation (Yandrasits et al., 2019). Recently, Liu and coworkers examined the scaling behavior of the conformations and dynamics of mesoscopic models of hydrated Nafion membranes as well as a polymer melt. The authors compared the results obtained from five distinct DPD parameterizations of hydrated Nafion including one based on electronic structure calculations and others mapped from Flory-Huggins parameters along with varying harmonic spring bonded interactions (Liu et al., 2018b). The effect of the chain stiffness was also investigated in the dry and hydrated Nafion systems. The increase in chain stiffness gives rise to larger polymer end-to-end relaxation time along with slow segmental dynamics due to the stronger back scattering in the velocity autocorrelation function and larger residence time within the “cage” formed by the nearest neighbors. To demonstrate detailed information concerning the ionic aggregates, quantitative distance-based cluster analysis with realistic microscopic images colored by unique IDs was further developed to provide a wealth of information on water-filled ionic channel size, shape, and connectivity (Liu et al., 2018a). The water cluster size distribution suggests that all five DPD parameterizations of Nafion correctly show a percolating network after a certain degree of hydration (Liu et al., 2018b). The percolation threshold of the water domains in Nafion was determined to occur at a hydration level of 5 H_2_O/SO_3_H through direct cluster analysis in contrast to a methodology that derived from radial distribution functions and the Connolly surface analysis (Liu et al., 2018a). Agglomerate of the small water domains is clearly demonstrated *via* this cluster analysis method (shown in Figure 3). These results further demonstrated the validity of a density functional theory (DFT) based parameterization (Sepehr and Paddison, 2016) to compute the interaction parameters needed for the repulsion interaction in the simulations. This scheme addresses the challenge for the charged beads without reducing the efficiency of the DPD methodology by implicitly considering electrostatic interactions for the charged beads. A non-empirical fragment molecular orbital (FMO) method (Okuwaki et al., 2018a; Okuwaki et al., 2018b) was also developed as a novel scheme to consider polarization and charge transfer between beads. It is interesting to note that DPD is flexible for hydrated ion exchange membranes in terms of parameterization and coupling with smoothed particle hydrodynamics (SPH) (Jorn and Voth, 2012) or a dissociable Morse potential between the protons and the base and/or water beads (Lee et al., 2016; Vishnyakov et al., 2018).

**FIGURE 4:**
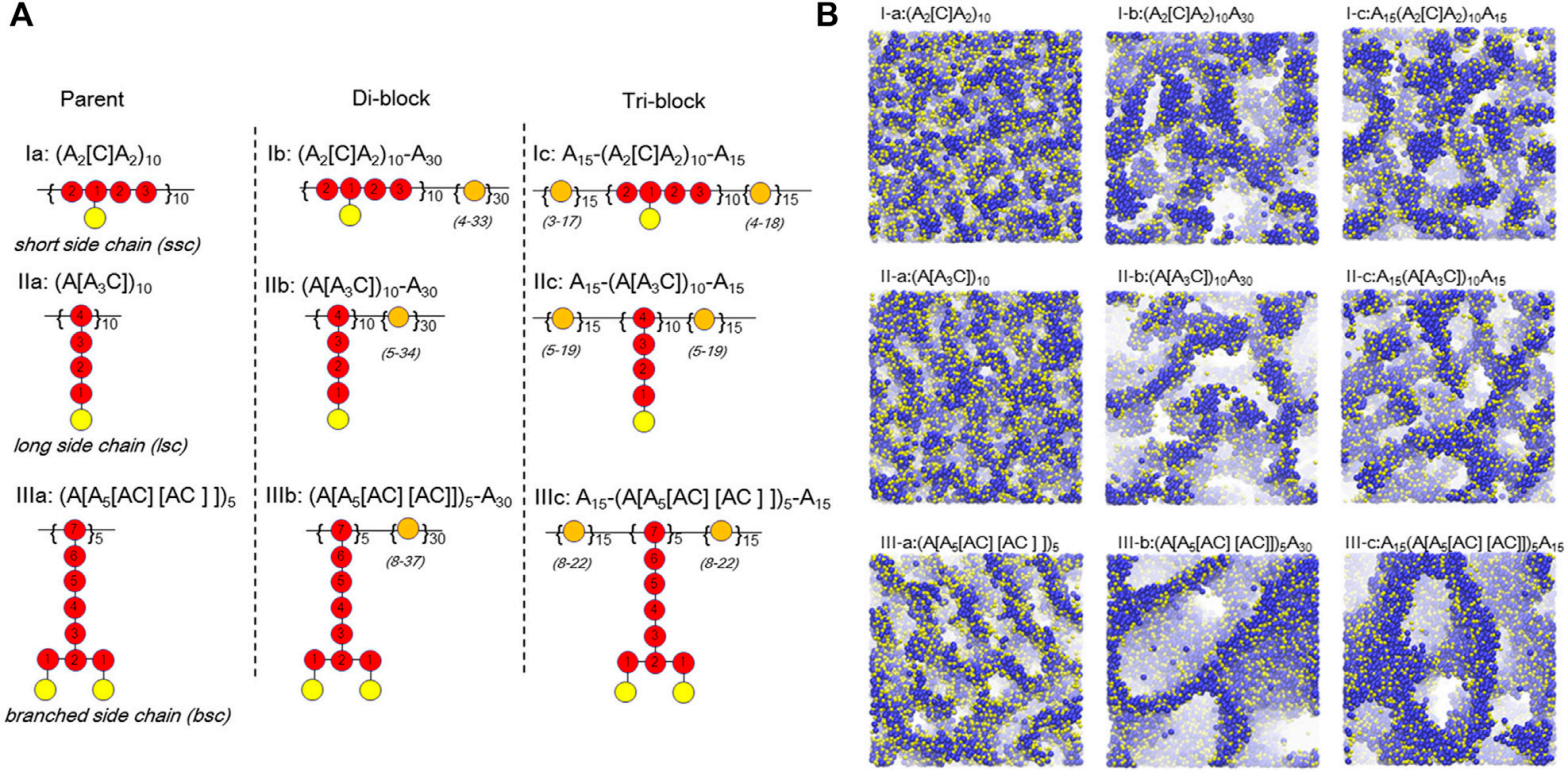
**(A)** The architectures of polymer chains including: amphiphilic parent (I-a; II-a; III-a), amphiphilic-hydrophobic di-block (I-b; II-b; III-b), and hydrophobic-amphiphilic-hydrophobic tri-block (I-c; II-c; III-c) functionalized with short side chain, long side chain, and branched side chain, respectively. Color codes: red, amphiphilic block beads; orange, hydrophobic block beads; yellow, C beads. Number of bonds, N_bond_, towards the nearest C bead Numbers is indicated within A beads. The range of N_bond_ for A beads within the hydrophobic block(s) is shown in parenthesis. **(B)** Simulated pore morphologies for corresponding architectures in **(A)**. Only hydrophilic beads are displayed. Color codes: blue, W beads; yellow, C beads. Reprinted and adapted from ref. (Dorenbos, 2017b). Copyright 2017 American Institute of Physics.

Based on the successful application of DPD simulation on PFSA-type PEMs, there have been several attempts to control the architecture of the backbones and side-chains. Dorenbos (Dorenbos, 2017b) recently constructed three different parent architectures of a backbone by covalently bonding hydrophobic beads ([A]) to: short hydrophilic beads ([C]); long ([A_3_C]) beads; or symmetrically branched A_5_[AC][AC] side-chains. Subsequently, three di-block copolymers and three tri-block derivatives were modeled by connecting an A_30_ block (30 hydrophobic A beads) or an A_15_ block with three different parent architectures as shown in Figure 4A. The results demonstrated that the increase in side-chain length enlarges the pore size for polymers of similar ion exchange capacity (IEC) and that the largest pore size appears in the systems with branched side-chains (Figure 4B). Meanwhile, the author investigated the effects of doping (A_2_[C])_10_ polymers with high IEC into a host polymer of A_30_(A[A_5_[AC][AC]])_5_ with a low IEC (Dorenbos, 2017a). These results show that the clearly distinguished pore networks disappear as the ratio of dopant is increased. This suggests that for a low dopant content the small amount of high IEC dopants is situated near well-connected pores formed by the low IEC host polymers, implying that one can increase the overall IEC of the blended membrane without sacrificing the percolated networks by adding a dopant. To further study the influence of designs with hydrophobic side-chains on the connectivity of the water containing domains, Dorenbos correlated the pore connectivity to the average number of bonds separating A from the nearest C ( $N_{bond}^{A-C}$ ) and between nearest C beads ( $N_{bond}^{C-C}$ ) (Dorenbos, 2019; Dorenbos, 2020). It was determined that the connectivity of the water domains generally increases with increasing $N_{bond}^{C-C}$ for the cases where $N_{bond}^{C-C}>N_{bond\left(\max\right)}^{A-C}$ and fixed IEC, while the opposite trend occurs for the cases where $N_{bond}^{C-C}<N_{bond\left(\max\right)}^{A-C}$ .

These results show a larger pore size for branched side-chain membranes than single side-chain membranes. Another potential strategy is to utilize multi-acid side-chains to improve the conductivity while retaining the good mechanical properties of the perfluorinated backbone, such as perfluorosulfonic imide side-chain perfluorinated polymers (PFIA) (Kusoglu et al., 2020) and perfluoroalkyl ionene chain-extended ionomers (PFICE) (Su et al., 2019). Compared with the PFSA material, the PFIA demonstrates higher proton conductivity at low hydrations due to both inter- and intra-side-chain interactions. The experimental and simulation results indicate highly ordered backbone configuration and better-dispersed smaller water clusters in PFIA than in PFSA (Kusoglu et al., 2020). Similarly, phase-separated morphology with enhanced local order was revealed by resonant X-ray scattering in both dry and hydrated PFICE ionomers, which improves proton dissociation at low hydration that can be discerned by the DFT-computed spectra of the PFICE side-chain (Su et al., 2019).

In addition to these modifications of the polymeric chemistry, the interactions between polymer segments and solvent molecules also have a strong influence on the aggregate structures. For instance, the aggregate structure of PFSA ionomers can be tuned with different solvents or mixtures of solvents (Welch et al., 2012; Doo et al., 2018). Fully atomistic MD simulations instead of coarse-grained approaches were used to study the effects of solvents (i.e., with dielectric constants, $\varepsilon_{\text{r}}$ = 2.38–109) in PFSA ionomer dispersions (Tarokh et al., 2020). The authors introduced a new aggregation phase diagram for Nafion in the various solvents. The ionomer chains are physically cross-linked through strong electrostatic interaction between ionic clusters in low dielectric solvents (i.e., $\varepsilon_{\text{r}}=1.41-42.5$ ), self-assembled into lamella-like aggregates *via* weak hydrophobic interactions in the solvents with intermediate dielectric constant (i.e., $\varepsilon_{\text{r}}$ = 42.5–78), and formed elongated aggregates because of strong hydrophobic interactions in high dielectric solvents (i.e., $\varepsilon_{\text{r}}\geq 78$ ). Simultaneously, the major deficiencies of a coarse-grained approach were revealed in that the aggregate structure in all solvents obtained from a coarse-grained model is unrealistic due to the fact that the solvent atoms are not explicitly modeled (Doo et al., 2018). More recently, experimental results show that the decrease in the polarity of the solvent increases the swelling of the fluoropolymer resulting in low proton mobility. Alkanes do not change the Nafion structure from its dry state due to the minimal interaction with fluoropolymer backbone and ionic groups (Katzenberg et al., 2021; Shi et al., 2021).

Compared to the pronounced hydrophobic/hydrophilic interactions in PFSA-based membranes, polymers with sulfonated aromatic rings have been explored as promising alternatives for fuel cell applications because they possess a wide variety of tunable chemical structures such as different degrees of sulfonation, various linkers between aromatic rings and fluorinated components of the macromolecules. Specifically, sulfonated Diels-Alder poly(phenylene) (SDAPP) (Abbott and Frischknecht, 2017; Clark et al., 2019; Sorte et al., 2019) and sulfonated poly(ether ether ketone) (sPEEK)(Chen S. et al., 2020) are the focus of many studies aiming at the elucidation of structure-property relationships. Frischknecht et al. (Abbott and Frischknecht, 2017; Clark et al., 2019; Sorte et al., 2019) undertook experimental and theoretical studies to investigate the influence of the degrees of sulfonation and hydration level as well as temperature on the morphology and the dynamic properties of water molecules and protons within SDAPP membranes (Sorte et al., 2019). The isolated hydrophilic clusters were observed at relatively low degrees of hydration and sulfonation and the increase of hydration and/or sulfonation improved the connectivity of the water (Clark et al., 2019). Besides the distance-based cluster analysis, the density-based clustering algorithm was applied to distinguish the local size and shape of the percolated water-containing clusters in particular for overlapped clusters. The clustering results obtained from these two algorithms demonstrated that the densest clusters were bridged by narrow regions. Furthermore, the relative anisotropy of the shape in these clusters quantitatively showed that the clusters became more spherical with increasing degree of hydration and sulfonation (Abbott and Frischknecht, 2017; Clark et al., 2019).

MD simulations show that sPEEK membranes possess stronger electrostatic interactions between the sulfonate oxygen atoms and hydrated protons leading to much lower hydronium ion diffusivity and lower delocalization of free H_3_O^+^ when compared to Nafion (Wang R. et al., 2019). The structure of hydrated hydronium ions deforms to adapt to the narrow regions of hydrophilic pores. The confinement effect on the diffusion of H_2_O and H_3_O^+^ is affected by the hydration level and the formation of conductive channels. It was concluded that weak nanophase separation and poor connected ionic domains in sPEEK membrane contribute to lower proton conductivity in comparison to Nafion. Okuwaki et al.(Okuwaki et al., 2018a) drew the same conclusion from FMO-DPD simulations of sPEEK and Nafion membranes.

As mentioned above, one conventional strategy is the blending of different types of polymers to enhance the chemical and physical properties of the materials. However, additional intermolecular interactions within the blends make the prediction of properties challenging. Recently, Ozbulut et al. studied the morphology of polymer blends of highly branched poly(arylene ether sulfone) (HBPAES) and linear poly(arylene ether sulfone) (LPAES) *via* both experiments and DPD simulations (Sevinis Ozbulut et al., 2020). The branched topology of HBPAES was tuned by changing the distance between the branch positions. The results revealed the miscibility of highly branched and linear polymers that was not influenced by the separation between the branch positions, which was further confirmed by the distribution of the radii of gyration for the pure polymers and the blend systems. It is also interesting to note that the increased strain at the break values is attributed to the anchoring regions rather than the strength of the non-bonded interactions in the blend.

Another promising route to advance the performance of non-PFSA membranes is to design highly ordered morphologies for efficient proton transport. This requires a fundamental understanding of how to balance the specific interactions within these materials. Recently, well-controlled chain folding has been achieved by Trigg and coworkers (Trigg et al., 2018) through precisely tethering sulfonic acid groups into the linear polyethylene backbone every twenty-first carbon atom to obtain highly ordered conductive layers for fast proton diffusion. The classical MD simulations further demonstrated that the diffusivities of water and hydronium ions were enhanced in the well-ordered water channels compared to that in the amorphous morphology. It was suggested that this state-of-the-art strategy may be applied for cation/anion transport as well as anhydrous proton transport by modifying the chemistry of functional groups.

### Hydrated AEM morphology

**FIGURE 5:**
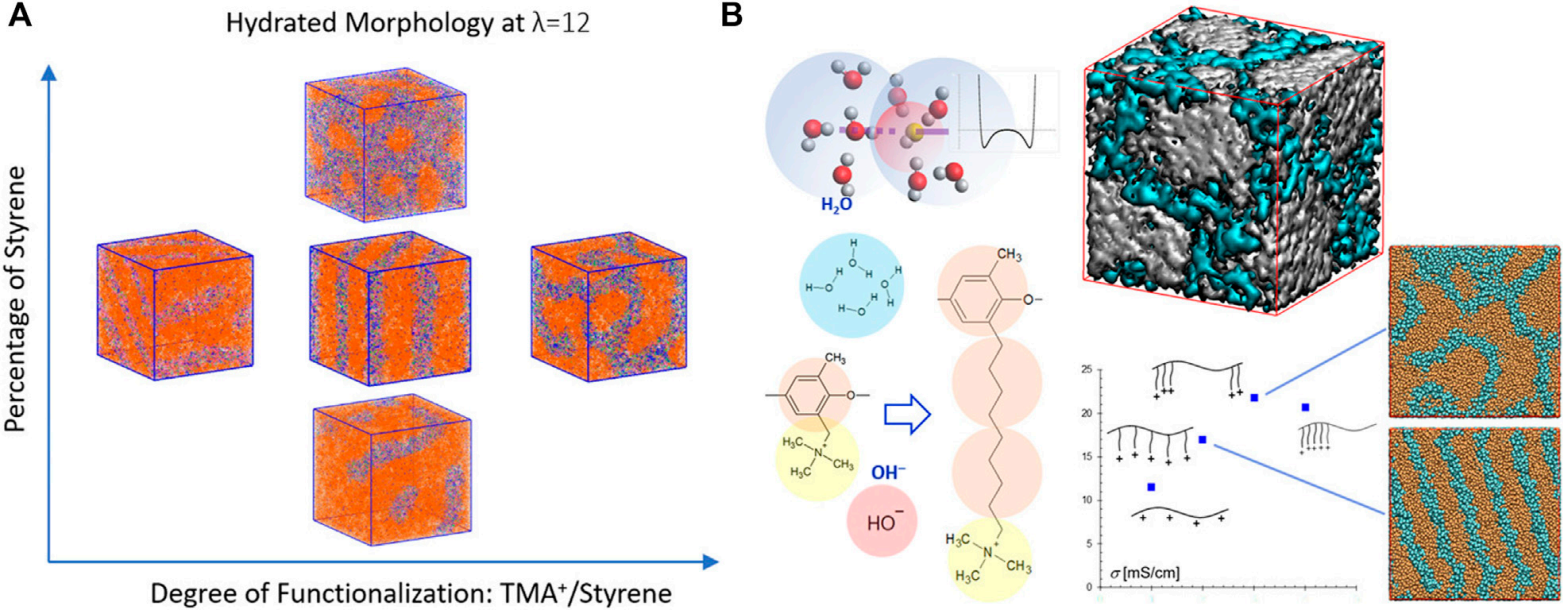
**(A)** Effects of the degree of functionalization and the content of polystyrene on the hydrated morphology of SEBS-based AEMs at λ = 12. Color scheme: orange, beads representing two polymerized vinyl groups; mauve, phenyl group; green, trimethylmethane group; purple, TMA; cyan, OH¯; and blue, water. **(B)** The hydroxide ion model and the Morse potential-based associative forces between hydroxide ion and water bead; molecular structures of the species and their coarse-grained models as well as the side-chain modifications of PPO-TMA; Simulated morphology of the hydrated PPO-C8Q-comb and PPO-C8Q-triblock at λ = 11. Color scheme: orange: polymer; and cyan: water. Isosurface of PPO-C8QC8Q-C16-triblock in which polymer domain and water domain are shown in white and cyan, respectively. Figure A reprinted with permission from ref. (Luo et al., 2020a) and figure B reprinted and adapted with permission from ref. (Lee, 2020). Copyrights 2020 and 2020 American Chemical Society.

In addition to PEMs, extensive investigations were performed on the hydrated morphology of polystyrene-b-poly(ethylene-co-butylene)-b-polystyrene (SEBS)-based AEMs (Sepehr et al., 2017; Luo and Paddison, 2019; Zhu et al., 2019; Luo et al., 2020a). The results indicated that the quaternary ammonium-functionalized SEBS AEMs phase separates into a functionalized polystyrene-rich phase and a hydrophobic phase. The water content clearly controls the morphology which evolves from perforated and interconnected lamella to perfect lamellae and then to disordered bicontinuous structures as the water content is increased (Sepehr et al., 2017). Reducing the functionalization and/or the percentage of styrene delays the formation of largely interconnected water networks (Figure 5A), but the increase in the styrene content facilitates the percolation to occur at a lower hydration level (Luo et al., 2020a). An alkyl spacer (C_4_H_8_) grafted between the backbone and functional QA group as the linker, tail, and as both a linker and a tail strongly affect the morphology, the domain spacing of the hydrophilic phase, the distribution of the backbone-TMA^+^ distance, and the water clusters. Specifically, additional spacers may postpone the occurrence of a percolated water domain and thereby evenly distribute the water (Luo and Paddison, 2019). The choice of the functional group (trimethylammonium, methylimidazolium, or trimethylphosphonium) was found to have minimum effect on the backbone structures while moderately affecting water distribution. Compared to the cationic groups, the associated anion seemed to have a greater impact on the size of the exclusive water domains (Zhu et al., 2019).

The influences of alkyl chain length, side-chain structure, hydrophilic and hydrophobic spacers, and distribution and aggregation of the side-chains on the microstructure of polyphenylene oxide tetramethylammonium (PPO-TMA) as well as SEBS-based AEMs were systematically explored with DPD simulations by Lee and coworkers (Lee, 2019; Lee, 2020; Lee, 2021). The comparison of PPO-based and SEBS-based triblock copolymers shows that the latter possesses more hydrophobic and flexible backbones, in which nano-segregation occurs even without any side-chain modifications. The comb-like structure distribution of the side-chains contributed to the formation of a lamellar structure. Short side-chains acting as spacers helped to break the percolated water domain into smaller domains as the cationic groups were close to PPO backbones, indicating the larger impact of an alkyl spacer than an alkyl extender. A lamellar morphology may be transformed into an interconnected water network by tailoring the pendant length or composition of the side-chains as shown in Figure 5B (Lee, 2020). Adding hydrophobic alkyl spacers was observed to enhance the phase separation and promote the formation of large water clusters. However, the strong hydrophobicity of the side-chains (either alkyl spacers or alkyl extenders) resulted in substantial protrusion into the water phase, which formed bottlenecks within the transport pathways retarding anion mobility. These results suggested that the less hydrophobic alkoxy spacers may help to form narrower but more connected channels (Lee, 2019). In contrast, adding hydrophilic spacers impedes the transport of hydroxide ions as well as water, ascribing to the stretched side-chains dispersing in the hydrophilic phase (Lee, 2021). This drawback may be resolved by altering multi-cation side-chain designs, which facilitate the formation of largely connected ion-conducting pathways. Coarse-grained MD simulations of two poly(ether ether ketone) (PEEK) membranes (Chen S. et al., 2020), in which the side-chains consist of either one or two quaternary ammonium (QA) groups, indicate that slightly smaller and better interconnected hydrophilic clusters occur in two QA than in single QA membranes and that the increase of grafting degree results in the decrease of cluster sizes for both membranes. In addition, the coordination number of OH¯ is greater in two QA systems than in single QA membranes, which probably improves the alkaline stability of two QA polymers (Chen S. et al., 2020). Furthermore, Li and coworkers (Li et al., 2021) found that both OH¯ conductivity and chemical/mechanical stability of the PPO-based AEM can be significantly improved by grafting a tri-cation side-chain with a long hydrophobic extender and an additional hydrophilic side-chain. These results further demonstrate the success of the strategy of multi-cations on each grafted functional group to balance the ionic conductivity and the chemical stability (Pan et al., 2013; Zhu et al., 2016; Li et al., 2021).

Alternatively, a delicate balance of hydrophilic and hydrophobic features in functional groups can mitigate the bottleneck issue (Zhang and Van Duin, 2015; Dong et al., 2018a; Dong et al., 2018c). Reactive force-field MD simulations were conducted on hydrated PPO-based homopolymers with five distinct quaternary ammonium cationic groups (R_1_: −CH_3_, R_2_: −C_2_H_5_, R_3_: −C_3_H_7_, R_4_: −C_6_H_13_ and R_5_: −C_4_H_8_OCH_3_) (Dong et al., 2018a). The hydrated morphology indicated that the nanoscale water domains were connected by narrow channels and the size of the water channels increased dramatically with increasing water content. For the systems with symmetrically modified cation groups, long alkyl tails (i.e., 3R_3_) enhanced phase segregation, giving rise to the formation of large water clusters with fewer connections between those clusters. For the systems with asymmetric cationic groups, the distribution of pore sizes was narrower in the 2R_1_R_4_ and 2R_1_R_5_ than in 3R_3_ at a hydration level of 5. Enhancement in the hydrophilicity for 2R_1_R_5_ by introducing an ether group resulted in a more uniform distribution of the size of the water channels when compared with 2R_1_R_4_, indicating the formation of narrow connections between the water domains. These findings show that the hydrophobicity of the functional groups should be strong enough to provide the desired nanophase segregation but not so strong as to provoke the formation of narrow bottlenecks between the water domains, e.g. 2R_1_R_4_ (Dong et al., 2018a).

Although a high IEC improves the ionic conductivity of an AEM, it also causes swelling of the membrane giving rise to low dimensional stability. One potential approach is to utilize cross-linking to manage excess swelling in AEMs. Experimental results have shown that cross-linking significantly reduces the water uptake and swelling without sacrificing the anion conductivity and the interdomain spacing of the AEMs decreases with the degree of cross-linking for the poly(bromopropyl norbornene)-b-poly(butyl norbornene) copolymers (Chen et al., 2019) and the TMA-functionalized SEBS membranes (Jeon et al., 2019) both by using *N,N,N*′*,N*′-tetramethyl-1,6-hexanediamine (TMHDA) as cross-linker, and the multiple quaternary ammonium-functionalized polysulfone AEMs modified with rigid β-cyclodextrin (Ma et al., 2020). However, few simulations have been undertaken to probe the influences of cross-linking on the morphology of AEMs despite the availability of the cross-link formation algorithm (Kacar et al., 2013).

To date, a significant effort has been devoted to determining rational design rules. Nevertheless, general conclusions have not been obtained since there is a complex interplay of a number of factors. To this end, we suggest that machine learning may be a promising approach to resolving the complexity of effects on the hydrated morphology of ionomers.

### Anhydrous morphology

In anhydrous ion-containing polymers, ionic aggregates form due to specific interactions including strong electrostatic, metal/ion-dipole, etc. The influence of polymer architecture, the dielectric constant, and the specific mobile ions on the morphology of the ionic aggregates are of significant interest. Similar to hydrated ionomers, only percolating ionic domains may serve as ion conducting channels thereby providing high ionic conductivity. Although experiments can provide microscopic images of the ionomer such as those obtained from scanning transmission electron microscopy (STEM), and the approximate ionic aggregate size distribution from X-ray scattering, the details of the morphology such as the shape, size, and the percolation of the ionic domains are incompletely understood (Ketkar et al., 2019). Thus, MD simulations have been undertaken to resolve this problem.

**FIGURE 6:**
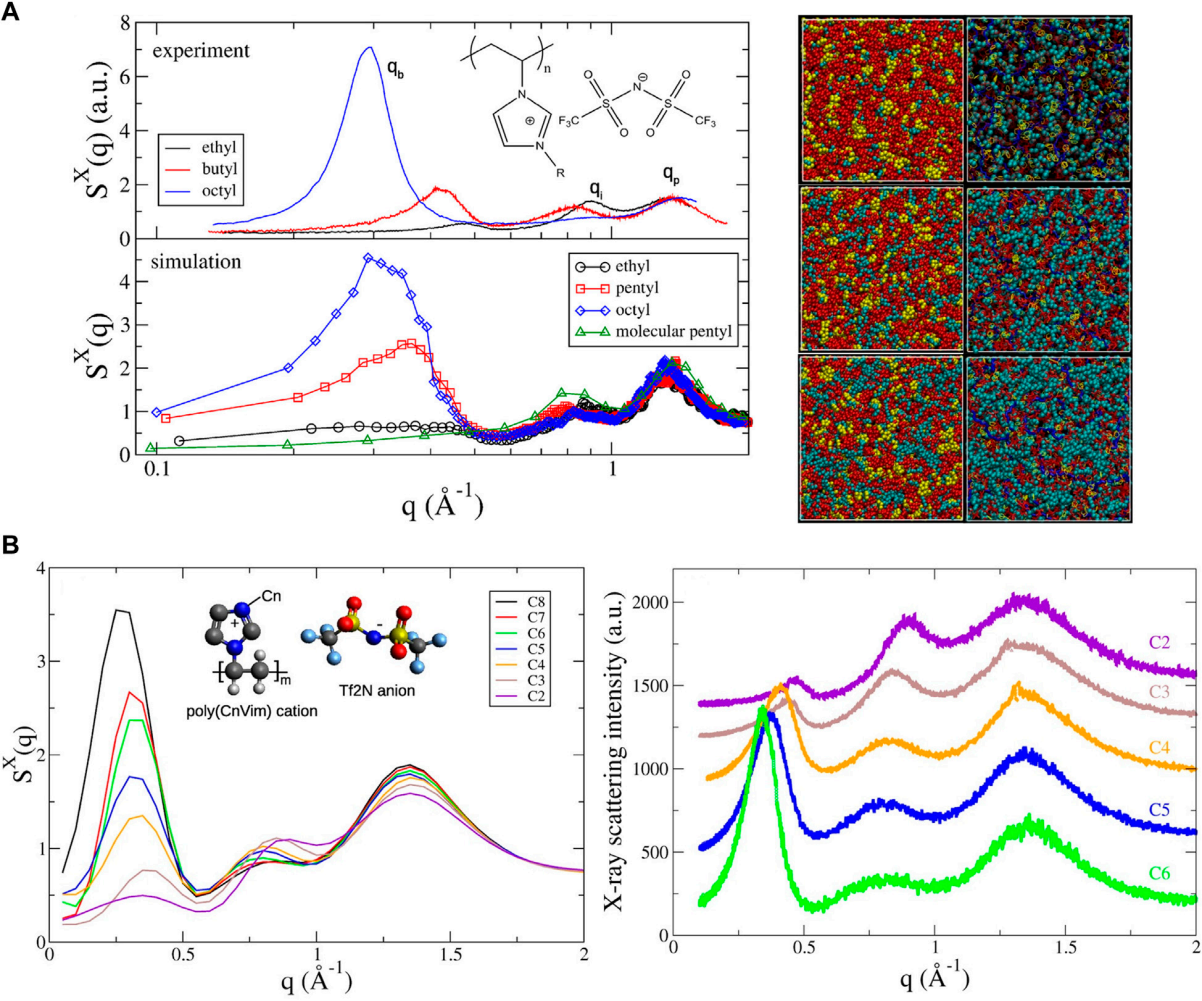
**(A)** Simulated X-ray scattering profiles of poly(CnVim Tf2N) as a function of alkyl side chain length (*n* = 2–8) compared with experimental data (left); morphologies shown with space-filling models as well as snapshots of poly(CnVim Tf2N) (n = 2, 5, and 8 from top to bottom) (right). Color scheme: red, anions; yellow, imidazolium rings; cyan, cationic tails; red bonds, anions; cyan sphere, cationic side-chains; yellow line, imidazolium rings; blue bonds, cationic backbones. **(B)** Simulated total X-ray structure factors with an inset of the molecular structure of poly(CnVim Tf2N) and experimental X-ray scattering profiles of poly(CnVim Tf2N). Figure A reprinted and adapted with permission from ref. (Liu and Paddison, 2016) and figure B reprinted with permission from ref. (Liu and Paddison, 2017). Copyrights 2016 and 2017 American Chemical Society.

Recently, Liu and Paddison performed atomistic MD simulations to study the structural properties of a homologous series of poly(n-alkyl-vinylimidzolium bistrifluoromethylsulfonylimide) (poly(nVim Tf2N)) ionic liquids (Liu and Paddison, 2016). The computed structure factors were observed to be in excellent agreement with X-ray scattering results in terms of peak position and shape. Three distinct peaks were characterized as the low-q peaks at 0.2–0.6 ${Å}^{-1}$ according to the backbone-to-backbone correlations ( $q_{b}$ ), the intermediated-q peaks in the range of 0.8–1.0 ${Å}^{-1}$ corresponding to the ionic interactions ( $q_{i}$ ), and the high-q peaks at 1.0–2.0 ${Å}^{-1}$ representing the pendant-to-pendant peak ( $q_{p}$ ) as shown in Figure 6A. The simulated nanostructures of poly(nVim Tf2N) (n = ethyl, pentyl, and octyl) on the right side of Figure 6A illustrated the dramatic growth of the nonpolar aggregates with increasing the length of alkyl chain along with the homologous series and was quantified by the distribution of the nonpolar cluster size. It is worth noting that the rational choice of the criterion for the connectivity in the distance-based cluster analysis algorithm is critical to correctly predicting the cluster size distribution. One strategy is to deconvolve the first peak of the radial distribution function by fitting it to a series of Gaussian functions and choosing the limit of the strongest Gaussian peak as the cutoff for the connectivity. Furthermore, a systematic increase in the alkyl chain length leads to the growth of nonpolar domain size (i.e., the backbone-to-backbone correlation length) and a slight increase in the ionic domains, despite a negligible shift of the pendant-to-pendant peak (Liu and Paddison, 2017). These results reveal a satisfactory agreement between simulation and experiment as shown in Figure 6B. The complex interplay between morphology and ionic conductivity is discussed in the following section.

Analogous to the hydrated morphology, nanophase separation has been observed in block copolymers containing polyILs experimentally by Elabd and coworkers (Ye et al., 2012; Choi et al., 2013) and by Ganesan group with simulations (Zhang et al., 2019; Zhang et al., 2021b). The results show that the lamellar block copolymer has a similar anion concentration in the center to that of the homopolymers while the random copolymer displays the lowest local ion concentration. Moreover, the strength of cation-anion coordination is influenced by introducing nonconducting monomers, in which frustrated coordination behavior occurs in the interfacial area of the lamellar block copolymer and the bulk regions of the random copolymer. This was rationalized to be responsible for the low anion mobility in comparison to the homopolymer (Zhang et al., 2019). It should be noted that a multiscale simulation framework consisting of CG modeling and reverse mapping of the CG morphology was developed to extend the length and time scales of the simulated systems (Zhang et al., 2021b).

Ionomers are another essential type of single-ion conducting polymers, in which only the mobile ions contribute to the conductivity. Ion transport is governed by the morphology of the ionic aggregates. In an effort to reveal the distinct morphologies consisting of these ionic aggregates that are included in the single ionomer peaks of X-ray scattering, Frischknecht and Winey (Frischknecht and Winey, 2019) performed microsecond-long, all-atom classical MD simulations on a series of precise poly(ethylene-co-acrylic acid) ionomers with mobile lithium ions at 423 and 600 K. The spacer lengths between the backbone and acid groups were varied and tuned. The percolated ionic aggregates were found in three spacer length systems that were totally neutralized with lithium ions. For partially lithium neutralized systems, the acidic groups prefer to stay on the sides or ends of the lithium ion aggregates with some coexisting acid aggregates formed *via* hydrogen bonding. The evolution of the ionic domains revealed that the morphology reached steady state over hundreds of nanoseconds at a higher temperature while it cannot within the microsecond-long simulation at a lower temperature.

In contrast, Agrawal and coworkers (Agrawal et al., 2016) demonstrated that the ionic clusters formed in PSS melts with both Na^+^ and Mg^2+^ did not percolate across the sample according to the definition of the cluster that consists of SO_3_¯ groups that are within the distance criterion of 6 Å. Specifically, the majority of the clusters in a PSS/Na^+^ system are highly ordered and ladder-like in shape, while the clusters in PSS with Mg^2+^ are irregular and elongated. The cluster size distributions for both melts illustrate that charge-neutral clusters with an even number of SO_3_¯ groups are dominant over charged ones. Although both melts have a broad distribution of cluster size, the PSS/Mg^2+^ has a larger average cluster size than that of PSS/Na^+^ even under shearing.

## Ion transport

Transport phenomena in ion-containing polymers comprise multiple mechanisms at different time and length scales. In hydrated ionomers, the rapid dynamical hydrogen bonding of the water facilitates the transport of ions in a vehicular (i.e., as a carrier) and/or in a structural way (i.e., typically in the case of proton transfer). Typically, ion diffusivity increases with hydration level, but a high degree of hydration generally causes swelling of the polymer matrix and may limit the operating conditions. In anhydrous polymeric systems, the ion conductivity is generally controlled by the slow segmental dynamics of the polymer. Thus, one strategy is to reduce the glass transition temperature ( $T_{g}$ ) or add liquid plasticizers, leading to the enhancement of the ion and segmental dynamics (Bocharova et al., 2017). In contrast, another method is to enhance the decoupling of ion transport from segmental dynamics (Wojnarowska et al., 2017), which would probably be achieved by the morphological confinement and/or tuning of specific interactions within ion-containing membranes.

### In hydrated systems

Hydrated membranes illustrate various hydrophilic ionic domains due to nanophase separation, in which various proton transport phenomena occur according to distinctions in the chemical structures of the ionomers. The formed hydrophilic ionic domains and in some cases, channels are composed of pendant functional groups, water molecules, and dissociated protons, giving rise to a complex confined environment for proton carrier transport.

Given the disparate range in the time and length scales of the phase-separated hydrated morphology and proton transport within a local environment, it is challenging with only a single model to properly describe proton transport (*via* structural diffusion) within a polymeric system and the conformation of a water network due to nanophase separation (Jorn et al., 2012). More importantly, the transport of H^+^ or OH¯ occurs through the migration of a charged defect in the hydrogen bond network through a sequence of elementary proton transfer reactions/events, which requires a quantum mechanical description of covalent and hydrogen bond breaking and forming (Tuckerman et al., 1997; Tuckerman et al., 2010). Thus, significant effort has been devoted to addressing these challenges. There are two major directions: simulating the diffusion of ions and/or water in an equilibrium morphology obtained from DPD simulations; and incorporating local interactions into coarse-grained MD/DPD simulations.

Dorenbos (Dorenbos, 2019; Dorenbos, 2020) performed grid-based Monte Carlo tracer diffusion calculations, which are capable of evaluating the diffusivity of water molecules based on equilibrium DPD morphologies but fails to describe proton transport behavior due to the lack of local interactions between the protons and the functional groups and solvent molecules. Another drawback is that the fixed pore networks cannot accurately represent the dynamical percolated water-containing domains (i.e., “channels”). A similar strategy was developed to simulate the transport of water in the fixed channels of the membrane (Johansson et al., 2015). The results obtained from both methods show that an increase in hydration improves the water diffusivity within the system (Johansson et al., 2015; Dorenbos, 2017a). It is interesting to note that the diffusivity of hydronium ions was also obtained from DPD simulations by modeling the hydronium ion bead with the proton associated with one to four water molecules (Xiao et al., 2018). The results indicate that the increase in the size of solvated hydronium complexes retards the hydronium transport.

Another approach is to map the coordinate of the coarse-grained water beads to the interpolation quasi-particles within the theory of SPH to describe proton transport in a mesoscopic structure of a PEM (tens of nanometers) (Jorn and Voth, 2012). This method combines the dynamic behavior of the protons from atomistic MD simulations and the mesoscopic structure of PEMs driven by the hydrophilic/hydrophobic interactions among the polymer matrix, functionalized side-chains, water, and protons. This scheme was applied to hydrated Nafion. To properly include double layer effects and the local strong interaction between the functional groups and protons, electrostatics and position dependent diffusivity were introduced. The computed conductivities showed good agreement with experimental data (Jorn and Voth, 2012). Moreover, proton transport behavior within various morphologies including lamellar, cylindrical, and cluster were also investigated using this SPH-based mesoscale method (Liu et al., 2015). The proton conductivity within the cluster morphology was found to be lower than that within the other two morphologies due to the higher porosity and tortuosity in the cluster morphology.

Apparently, these methods consider the proton/water transport indirectly based on the static equilibrated morphology. The dynamic dissociation and association of protons from the acidic groups cannot be simulated. An elegant way to incorporate protonation/deprotonation processes is to introduce a dissociative Morse potential into mesoscale DPD simulations. Neimark and coworkers (Lee et al., 2016; Vishnyakov et al., 2018) performed DPD simulations in synergy with dissociable Morse bonds between the protons and the conjugate bases and/or water beads to model proton hopping occurring in PEMs. This strategy successfully achieves the goal of simultaneously simulating the explicit proton/water transport and the nanophase separation in hydrated PEMs. Model Nafion membranes were simulated using this method and the resulting proton diffusivities at moderate and high hydration levels agreed well with the diverse experimental data. Nevertheless, there was a significant discrepancy at low hydration, which was ascribed to the dynamic narrow bridges between water domains that required a higher fidelity in the simulations than achievable in DPD simulations (Vishnyakov et al., 2018). Importantly, the results showed a sharp increase around the percolation threshold. This phenomenon was directly observed using PFG-NMR by Vasenkov et al. (Berens et al., 2020). It is worth noting that the dynamic hydrophilic subphase connectivity plays a critical role in water diffusion, suggesting that it is still challenging to accurately estimate proton diffusivity at low hydration through coarse-grained modeling.

In an effort to mimic the structural diffusion of protons, various techniques have been implemented into classical MD simulations including multistate empirical valence bond models (e.g. MS-EVB) (Savage et al., 2014), bond order-based reactive force fields (e.g. ReaxFF) (Zhang and Van Duin, 2015), and quantum hopping (Q-HOP) (Devanathan et al., 2010). Recently, a combination of reactive and nonreactive polarizable MD simulations was used to study transport mechanisms of OH¯ in hydrated PPO-QA AEMs (Dong et al., 2018c). The morphology of these non-blocky AEMs at a hydration of 10 exhibited narrow percolating channels connecting the water domains. It was found that the vehicular mechanism made the dominant contribution to the conductivity in water-rich domains, while structural diffusion played a significant role in crossing the bottlenecks between large water domains under the confined environment of a channel (Dong et al., 2018a; Dong et al., 2018c). Their findings demonstrate that structural diffusion of OH¯ facilitates the diffusion through these bottlenecks without loss of anion hydration structure, while the vehicular diffusion of OH¯ through the bottlenecks requires a change in the hydration structure giving rise to a high kinetic barrier for such events. Notably, the decomposition of the total diffusivity into vehicular motion and discrete structural diffusion revealed that the structural diffusion is dominant and the structural diffusion is anticorrelated with the vehicular diffusion (Dong et al., 2018b).

To capture the delicate interplay of proton transfer and solvation structure, AIMD simulations are utilized, in which “on the fly” force fields obtained from DFT-based electronic structure calculations enable one to accurately simulate the breaking and formation of covalent bonds. However, the extremely high computational cost of AIMD simulations limits the accessible length and time scales. Thus, it is important to make careful selection in the simulated systems. Recent AIMD simulations of a model PEM functionalized with sulfonate end groups (SO_3_^−^) using graphane bilayers were undertaken to study the hydronium ion diffusion mechanism (Zelovich et al., 2021; Zelovich and Tuckerman, 2021). Surprisingly, low coordination of the hydronium ions at a hydration level of 3H_2_O/SO_3_H promotes the participation of the pendant SO_3_^−^ groups in the structural diffusion process of the hydronium ions with the reaction: SO_3_¯ + H_3_O^+^ ↔ SO_3_H + H_2_O. It was reasoned that the non-uniform water distribution at a low hydration level facilitates the reaction between the hydronium ion and the anion group as the oxygen located next to SO_3_¯ obtains a coordination number of approximately one from surrounding H_2_O and H_3_O^+^ (Zelovich et al., 2021). Furthermore, the sulfonate functional group with the linker of (CH_2_)_2_ possesses weaker acidity than with the linker of (CF_2_)_2_, resulting in more active participation in the SO_3_¯/H_3_O^+^ reaction and thereby improving instead of impeding the hydronium transport (Zelovich and Tuckerman, 2022).

**FIGURE 7:**
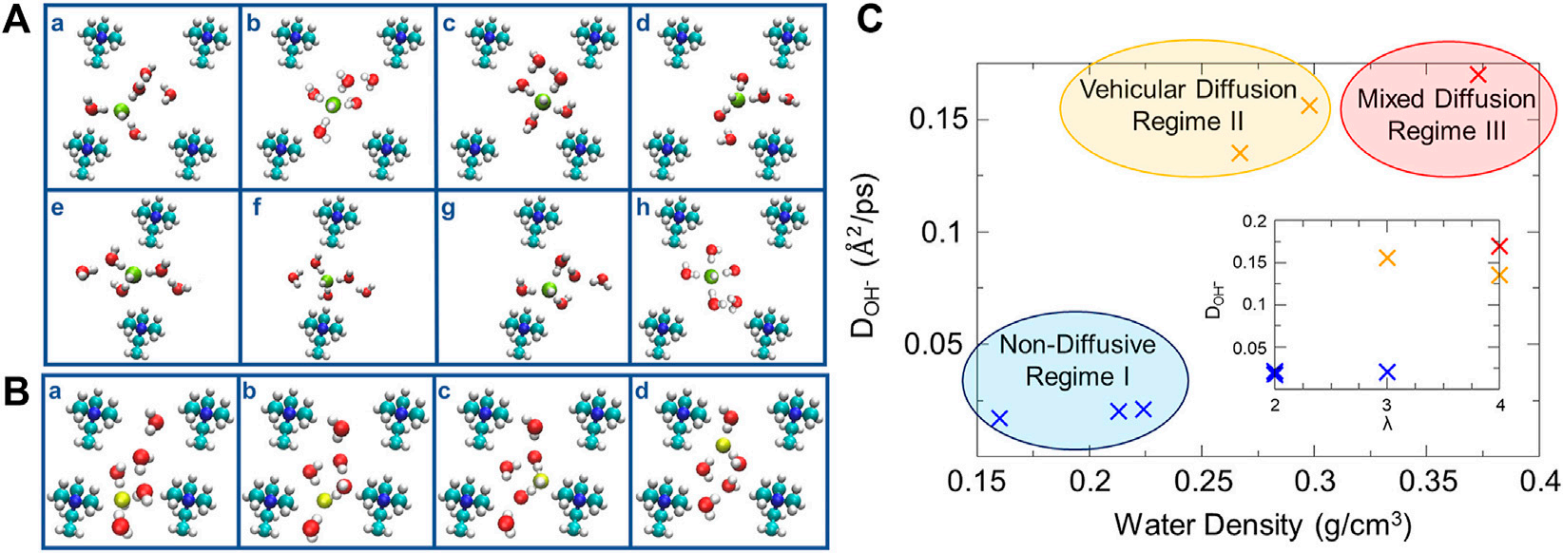
**(A)** Vehicular diffusion mechanism of OH¯ in system a4 from a z-perspective. **(B)** Structural diffusion mechanism of OH¯ in system b4 from a z-perspective. **(C)** Relationship between average diffusivity of OH¯ and water density, inset: OH¯ diffusivity vs. the hydration level. Reprinted and adapted with permission from ref. (Zelovich et al., 2019b). Copyright 2019 American Chemical Society.

Zelovich and coworkers (Zelovich et al., 2019a, Zelovich et al., 2019b) also employed graphane bilayers or carbon nanotubes with selected cationic groups attached with added water molecules and hydroxide ions to model the complex nanoconfined environment of an AEM. The effects of the hydration level, cation spacing, and cell geometry were studied to explore the mechanisms of OH¯ transport. Structurally similar water layers exhibit a symmetrical distribution of oxygen atoms between the graphane layers while structurally dissimilar water layers enable the flow of water molecules. These exist in different systems under various structural confinement. The structural similarity in the water layers reduces the hydroxide ion diffusion, while structural dissimilarity improves the hydroxide ion transport. In regard to enhancing OH¯ diffusion, various solvated OH¯ structures were considered to be active/inactive complexes. The active 3-fold and inactive 4-fold structures (i.e., hydroxide ion coordinated by three or four water molecules) correlate with fast and slow time scale processes, respectively, based on the fitting of results from proton transfer correlation functions. In systems of low hydration, the cation stabilizes the 3-fold complex acting as a fourth neighbor (shown in subplot a of Figure 7A), followed by the drift of this 3-fold complex toward the water molecules in the center of the cell forming a 4-fold planar structure (shown in subplot c of Figure 7A). This complex continues to move toward the nearby cation forming a stable 3-fold structure in the vicinity of the cation. An insufficient number of water molecules around the hydroxide ions leads to vehicular movement of the 3-fold complex between cationic groups. Increasing hydration helps build up a wire between the cation groups by rearranging the hydrogen bonds, which consequently invokes a series of proton transfer events *via* structural diffusion (shown in Figure 7B). Three distinct diffusion regimes were identified under low-hydration conditions: a non-diffusive regime at very low effective water density, a vehicular diffusion regime at intermediate water density, and a mixed diffusion regime with higher water density (Figure 7C). In contrast to PEMs, the cationic groups in AEMs form a bottleneck-like region for hydroxide ion transport at low water contents, consequently giving rise to the suppression of the diffusion of hydroxide ions (Zelovich et al., 2019a; Zelovich et al., 2019b; Zelovich and Tuckerman, 2021). Moreover, it is surprising to observe the non-monotonic temperature dependence of hydroxide diffusivity in both theoretical and experimental studies (Zelovich et al., 2022). This unexpected phenomenon was rationalized by examining the transport mechanisms of hydroxide ions at different hydration levels. Specifically, the vehicular diffusion is dominant at extremely low hydration (*λ* = 2) and high temperature is required to break the hydrogen bonds of the stable solvated hydroxide clusters. At low hydration (2 < *λ* < 5), moderate temperatures enhance trivial proton rattling but the hydroxide ions are still trapped in the cell center, which requires high temperatures to overcome the bottleneck between cations. Layered water structures occur at high hydration (*λ* > 5), leading to higher hydroxide diffusivity in the upper layer than in the cation-containing layer. Therefore, the temperature threshold for high hydroxide diffusivity occurs when the hydroxide ions can move to the upper layer where efficient structural diffusion is predominant (Zelovich et al., 2022). It may be concluded that the water distribution and the solvation structure of OH¯ play a key role in determining the proton transport mechanisms. Obviously, the combination of water density, the space between cationic groups, and confinement conditions results in complex local environments.

Recognizing the important role of the solvation structure of OH¯ under functionalized nanoconfined conditions, further study of the interplay of solvation patterns and diffusion mechanisms was undertaken by Zelovich and Tuckerman (Zelovich and Tuckerman, 2020). Five layers were defined and distinguished as outer water layers (i.e., the water in immediate proximity to the graphane bilayer L1 and L5), and inner water layers between the outer ones based on the distribution of the oxygen atoms. Planar 3-fold and 4-fold solvation structures were observed in L1 and L5, respectively. This leads to a less preferable environment for enhancing hydroxide diffusion due to the relatively stable planar structures. The inner water layers exhibit the solvation characteristics similar to that in bulk aqueous hydroxide. Specifically, in the L2 water layer located on top of the cationic groups, a series of successive proton transfer events occur followed by a quiescent period, while the frequency of proton transfer in the L3 and L4 layers is higher than in the L2 layer. These interesting results show a novel avenue to design high-performance AEMs by engineering the water within a nanoconfined environment.

The transport mechanisms of hydroxide ions within poly(arylene ether sulfone ketone)s functionalized by quaternized ammonio-substituted fluorenyl groups (QPE) AEMs were investigated *via* classical and first-principles MD simulations (Takaba et al., 2017). The impact of the number of repeating units in QPE on the ion conductivity of OH¯ is minimal and OH¯ transport in hydrated QPE occurs through structural diffusion (i.e. hopping between the ammonium groups of QPE) accompanied by vehicular transport based on the classical MD results. The first principles MD simulations show that the transport of OH¯ occurs through the formation of H_3_O_2_¯ *via* the hydrogen bond network of the water molecules. AIMD simulations of the quaternary ammonium functionalized polystyrene-block-poly(ethylene-ran-butylene)-block-polystyrene (QSEBS) AEMs systems demonstrate that hydration increases structural diffusion, which contributes to OH¯ conductivity, and hydroxide ions have the longest lifetime when the system is dry (Castañeda and Ribadeneira, 2020).

### In anhydrous systems

In addition to extensive investigations of proton transport in aqueous polymeric electrolytes, the mechanisms underlying ion transport in polyILs because of their application in batteries have attracted attention. Significant effort has been undertaken to address the low conductivity in polyILs which may be promising materials due to their long lifetime and safety advantages. Thus far, many factors have been studied to promote a better understanding of ion transport mechanisms and formulate design rules for high-performance polyILs (Aziz et al., 2018; Bocharova and Sokolov, 2020; Son and Wang, 2020).

**FIGURE 8:**
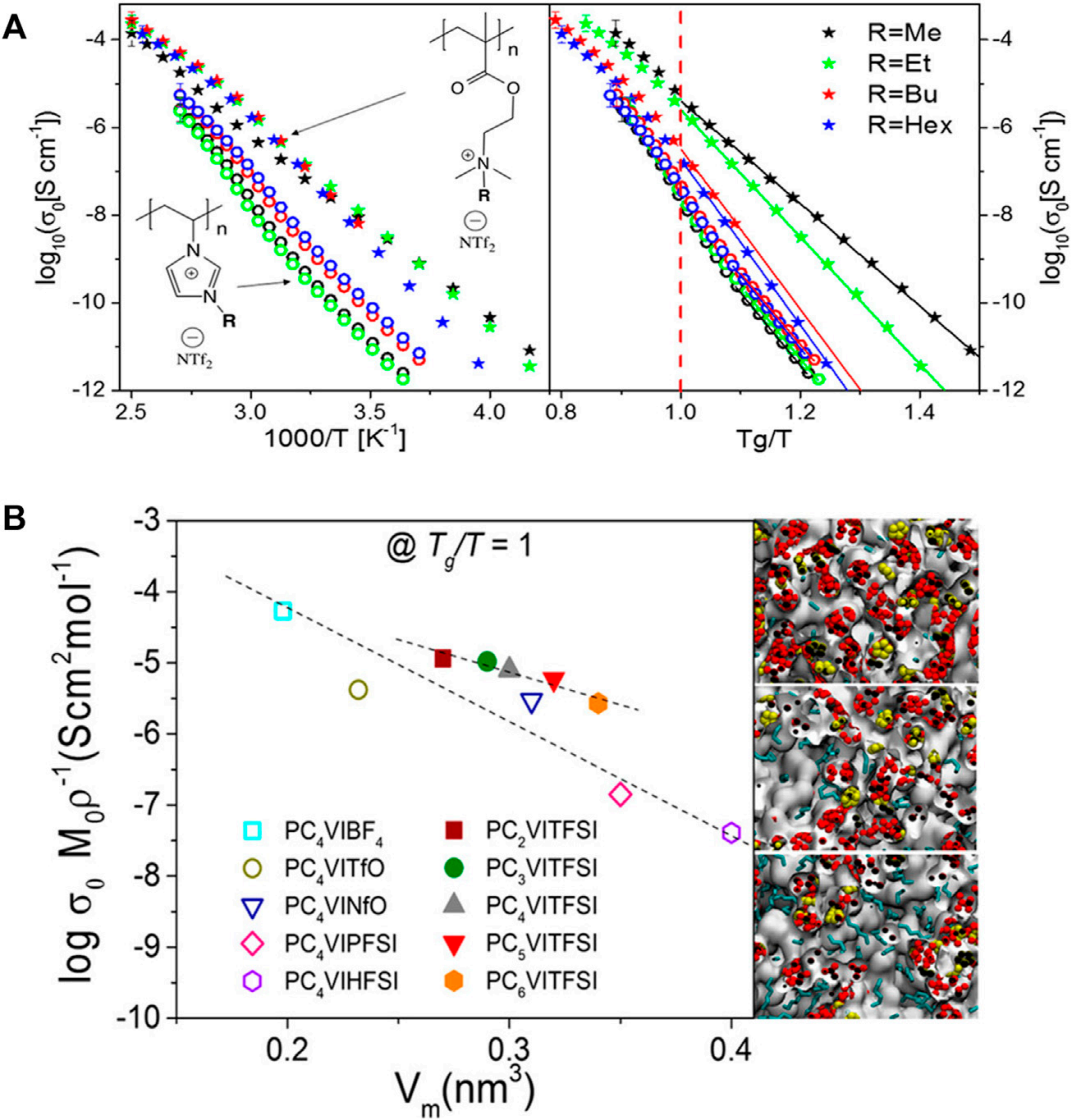
**(A)** Left: dc ionic conductivity vs. inverse temperature; right: T_g_-independent ionic conductivity for ammonium (stars) and imidazolium (open circles) based polyILs. **(B)** Anion concentration-normalized dc conductivity at T_g_/T = 1 as a function of repeat unit molecular volume, V_m_, (including the counterion) for imidazolium-based polyILs with different side chains and counterions. Figure A reprinted with permission from ref. (Heres et al., 2018) and figure B reprinted with permission from ref. (Iacob et al., 2017). Copyright 2018 and 2017 American Chemical Society.

Early studies (Iacob et al., 2017; Heres et al., 2018) revealed that the length and rigidity of the side-chains play a crucial role in transporting ions along the polymer chains. It was found that ammonium-based polyILs show higher dc ionic conductivity than imidazolium systems at their respective glass transition temperatures based on the results from BDS, WAXS, and classical MD simulations (shown in the left panel of Figure 8A) (Heres et al., 2018). Additionally, the alkyl spacer length has a strong influence on the ionic conductivity at comparable time scales of the segmental dynamics for imidazolium polyILs (shown in the right panel of Figure 8A). Specifically, in the imidazolium polyILs, the *T*_*g*_-independent ionic conductivity experiences one decade decrease as the alkyl side-chain length is systematically increased, which is correlated with an increase in the characteristic backbone-to-backbone distance (Iacob et al., 2017). In contrast, the reduction of anion molecular volume leads to ∼3 orders of magnitude increase in the *T*_*g*_-independent ionic conductivity. Complemented by atomistic MD simulations, the morphologies were transformed from isolated apolar clusters within a continuous polar matrix for shorter alkyl groups to bicontinuous sponge-like morphologies for longer alkyl chains, which is shown in Figure 8B. Variation in the molecular structure results in greater effects on ion mobility than the effective number density of the mobile ions (Heres et al., 2018).

**FIGURE 9:**
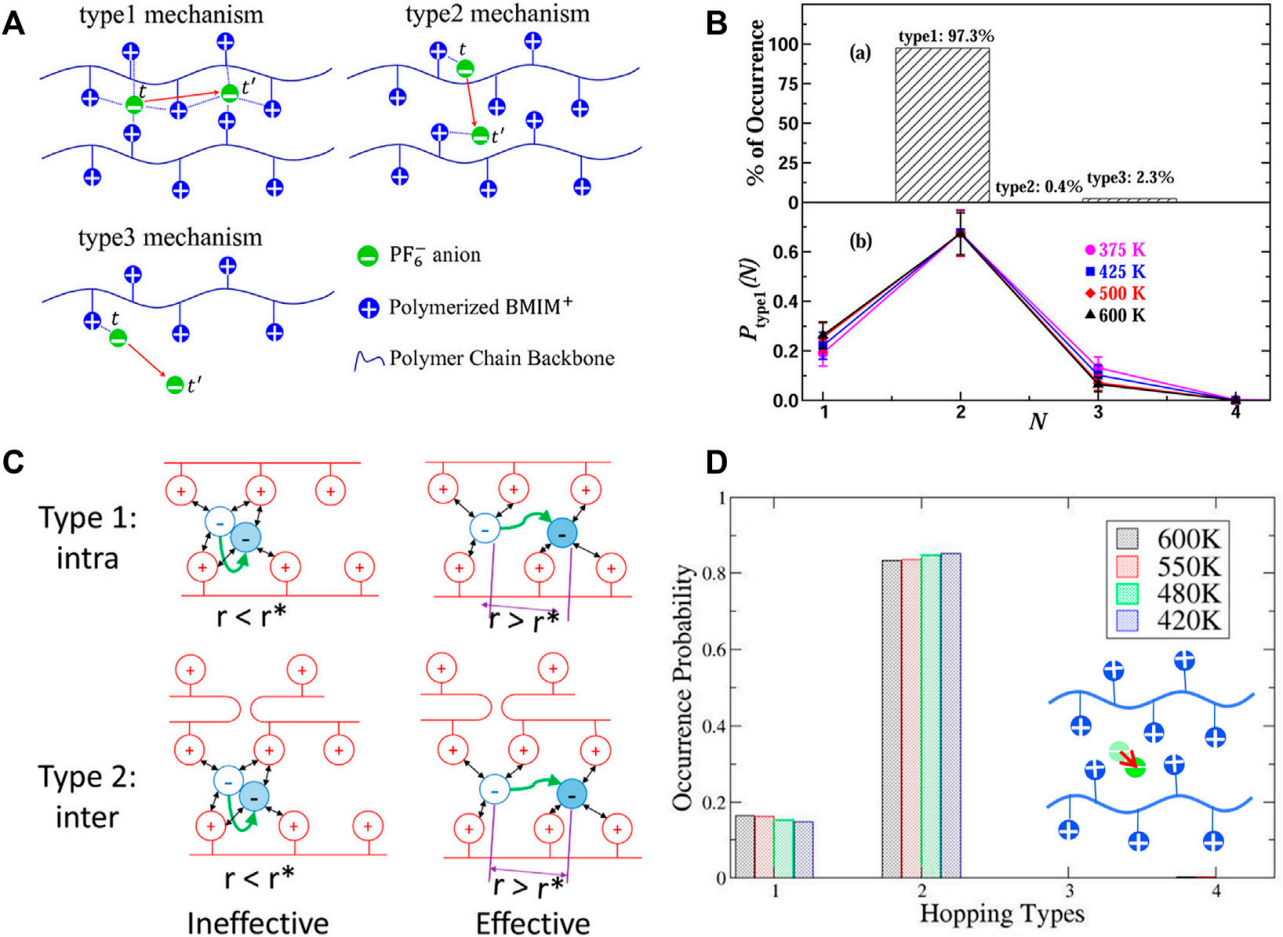
**(A)** Illustration of ion hopping types in polyIL electrolytes. **(B)** Percentage of hopping events in polyIL electrolytes (top); and decomposition of intrachain hopping events (type 1) assisted by N number of polymer chains (bottom). **(C)** Definition of effective hopping contributing to the long-range diffusion. **(D)** Probability of occurrence as a function of hopping type at four different temperatures when the ineffective ions were excluded. The inset is the definition of type 4: remaining intact (i.e. rattling). Figures A and B reprinted with permission from ref. (Mogurampelly et al., 2017) and Figures C and D reprinted and adapted with permission from ref. (Liu et al., 2021). Copyrights 2017 and 2021 American Chemical Society.

Ganesan et al. used atomistic MD simulations based on quantum-mechanically parametrized force fields to obtain a molecular-level understanding of the time scale and mechanisms underlying ion transport in model systems of poly(1-butyl-3-vinylimidazolium-hexafluorophosphate) polyILs (Mogurampelly et al., 2017). The intermittent and continuous correlation functions were used to probe the structural relaxation behavior of the association of anions and cations, and the average lifetime of the associations. The former was calculated with C(t)=h(t)h(0)/h , where *h*(*t*) is unity if one ion remains associated with another according to the first minima of the cation-anion radial distribution function at a given time *t*. The structural relaxation time was extracted by fitting the function to a stretched exponential. Alternatively, the structural relaxation processes can be captured from the intermediate scattering function at a characteristic wave vector, corresponding to the first peak of the anion-anion radial distribution function. Based on these detailed analyses, their results show that anion transport in polyILs is mainly governed by a mechanism involving intra- and inter-chain ion hopping that is facilitated through the breaking and formation of ion associations involving four polymerized cationic groups that belong to two polymer chains (*see* Figures 9A,B). This finding was confirmed by atomistic simulations that included polarizability effects (Zhang et al., 2022). More interestingly, the authors found that the mobile ions tend to associate with three polycations from two polymer chains when refining the ion hopping mechanism by excluding the rattling ions (Zhang et al., 2022). They determined that ion mobilities in these imidazolium-based polyILs are directly correlated to the average lifetimes of the ion associations while the ion mobilities in pure ILs are correlated to the structural relaxation time; indicating that anion hopping along the cation monomer chains underlies the transport of ions in polyILs (Mogurampelly et al., 2017). These results conclusively imply that anion transport is decoupled from the slow segmental relaxations in imidazolium-based polyILs, which is different from what has been observed in poly(ethylene oxide) and poly(propylene glycol) systems (Yoshida et al., 2011; Wang et al., 2014). The difference may be ascribed to the rigid polymer matrix that facilitates decoupling of ion transport from segmental dynamics.

However, it was subsequently argued that the breaking and forming of ion association probably do not contribute to long-range ion transport because most ions merely “rattle” amongst the polymerized cationic monomers within a cage (Xiao et al., 2020; Liu et al., 2021). No long-range transport may occur unless the ion escapes from its cage. Based on the MD simulations performed on both 1-butyl-3-methylimidazolium tetrafluoroborate ([BMIM]-[BF4]) IL and poly(1-butyl-3-vinylimidazolium-tetrafluoroborate) (poly([BVIM]-[BF4])) polyIL, the comparison between ion transport in ILs and that in polyILs confirmed that the anion diffusivity in polyIL is directly correlated with the anion hopping among cages formed by the cationic branch chains. Hence, the trap time in which an anion is trapped inside a cage determines the time scale of the anion transport rather than the ion association lifetime (Xiao et al., 2020). Nevertheless, the coupled motion of an anion and the surrounding ions governs the diffusivities of ions in the IL, i.e., the ion transport is correlated with the ion association lifetime. These phenomena were observed in additional all-atom MD simulations (Luo et al., 2020b; Liu et al., 2021), which improved the understanding of ion hopping mechanisms in polyILs. Specifically, the ion transport mechanism in polyIL poly(C2Vim)Tf_2_N was investigated by defining an additional ion transport mode, i.e., an intact type that corresponds to the rattling of an ion when trapped in a cage. As expected, there is a significant portion of intact anions (35–50%), implying that a considerable amount of mobile anions are trapped in the cages and do not contribute to long-range transport. Examination of the self-part of the van Hove function of the anions at the characteristic time of the greatest dynamical heterogeneity illustrates the occurrence of a secondary peak, which is a quantitative indication of diffusion *via* hopping (Liu et al., 2021). Note that the dynamical heterogeneity can be quantified with a non-Gaussian parameter ( $\alpha_{2})\;$ to indicate the deviation from purely Gaussian behavior and is required to be large enough ( $\alpha_{2}>1)$ to distinguish the double peaks in the self-part of the van Hove function. If the effective mobile anions are distinguished from slow “immobile” ones according to the distance criterion of the local minimum between the two peaks in the self-part of the van Hove function of the anions, the categorization for only these effective mobile anions shows negligible intact anions and dominant inter-chain hopping over intra-chain events. This is in contrast to the classification with a short interval of sampling (Figures 9C,D). Indeed, these results conclusively show that the instant ion dissociation is necessary but not sufficient for effective hopping transport. Furthermore, the stringlike cooperative motion of the mobile ions was quantified according to the criterion: $min\left[\left|r_{i}\left(t\right)-r_{j}\left(0\right)\right|,\;\left|r_{i}\left(0\right)-r_{j}\left(t\right)\right|\right]<r_{s}$ , in which $r_{i}\left(t\right)$ and $r_{j}\left(t\right)$ are the coordinates of *i*th and *j*th anions at time *t*, and $r_{s}$ is the cutoff distance for the string collection. The average string length for the polyILs is in the range of 1.6–2.2 anions, but close loops in the anion transport were not observed, which were hypothesized to be the consequence of the low ionic conductivity in polyILs (Stacy et al., 2018).

A subsequent study (Luo et al., 2020b) provides some understanding of the ion transport mechanism in the polyILs with various counterions (Br¯, BF_4_¯, PF_6_¯, and Tf_2_N¯). It was further confirmed that only a small fraction of the anions contributes to effective hopping transport. Although the relationship between the diffusivity of anions and the lifetime of ion association is also close to unity based on the power law fitting, it was suggested that the linear correlation is more possible between the diffusivity of the anions and the structural relaxation rather than the lifetime of ion association. Additionally, these findings show that both the stronger interaction between cationic groups and Br¯ and the flexibility of Tf_2_N¯ hinder effective hopping, which implies the significance of specific interactions in polyILs governing transport mechanisms. Notably, the analysis methods employed in these studies including the categorization of hopping types, the definitions of dynamic heterogeneity and stringlike cooperative motion, can be extended to other ion-containing polymer systems.

**FIGURE 10:**
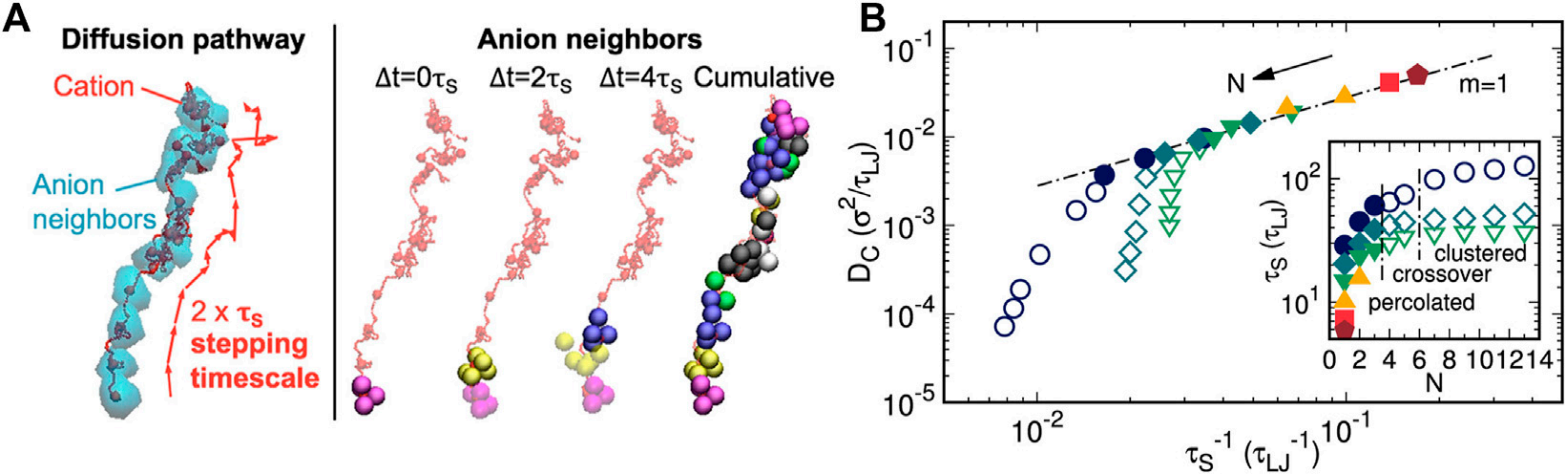
**(A)** Instant anion neighbor positions at the first three successive ∆t = 0 *τ*_*S*_, 2 *τ*_*S*_, and 4 *τ*_*S*_ increments, where transparent and opaque beads are previous and present neighbors, respectively. The three sets of anion neighbors (magenta, yellow, and purple) comprise no shared anion identities. **(B)** Cation diffusivity *D*_C_ vs. inverse characteristic ion-association lifetime *τ*_*S*_^−1^ for all spacer lengths *N* and dielectric constants ε_R_. Reprinted with permission from ref. (Bollinger et al., 2020). Copyright 2020 American Chemical Society.

In view of the role of ionic aggregate morphology in the mechanisms of ion transport, coarse-grained MD simulations were performed on single-ion conducting polymers to obtain an understanding of how the nanoscale aggregation of the cations and anions covalently bonded to the polymer affect the rates and mechanism of cation transport (Bollinger et al., 2020). The authors suggested that cations diffused *via* stepping motions along with the ionic aggregates and correlated the diffusivity of cations to the lifetimes of continuous association between oppositely charged ions in percolated ionic aggregate formed systems (shown in Figure 10). It was emphasized that this correlation did not hold for systems with isolated ionic clusters. Additionally, a strategy was proposed to enhance ion transport based on the strength of the Coulombic interactions: sufficiently strong to support percolated aggregates but weak enough to facilitate ion dissociation (Bollinger et al., 2020).

The mechanisms of ion transport are more complex in salt-doped polymeric ionic liquids due to a variety of interactions between the polymer and ions and between the ions themselves. It was found that an increase in salt concentration reduces the movement of ion species attributing to the increased viscosity upon the addition of a salt (Haskins et al., 2014). However, recent experimental and simulation results (Wang X. et al., 2019; Zhang et al., 2020a) have shown that in lithium salt-doped poly(1-butyl-3methyl-imidazolium bistrifluoroimide) systems, the addition of salt increases the dynamics of both lithium and the anions. It was also observed that there is a stronger coupling between lithium-ion transport and polymer segmental dynamics as well as the decoupling of the anion mobility from polymer segmental dynamics. The lithium ions show stronger dependence on salt concentration than the anions, which leads to higher lithium-ion transference numbers (Zhang et al., 2020a). Another surprising trend was recently deduced by Seo et al. (Seo et al., 2019). They determined that the increase in molecular weight enhances the ionic conduction in di-block copolymers using coarse-grained MD simulations, introducing an extra $1/r^{4}$ solvation potential. They suggested that the solvation strength dominantly controls the relationship between ion diffusion and ion concentration. This counterintuitive trend can be best explained by local ion agglomeration caused by the strong ion-ion interactions rather than interfacial width. Furthermore, the increase in molecular weight mitigates the ion agglomeration effect, consequently leading to high ion diffusivity due to a more even distribution of ions in salt-doped block copolymers.

To achieve high lithium selectivity (indicated by transport number and transference number) in polymer electrolytes, one potential approach is to incorporate nanoparticles into ion-conducting solid polymers or liquids; although the mechanisms underlying ion transport in such materials remain poorly understood (Diederichsen et al., 2017). Recently, coarse-grained multiscale simulations were performed on this type of polymer (Kadulkar et al., 2020). Their results suggest that the ionic conductivity is mainly due to Li^+^ transport along nanoparticle surfaces, in the vicinity of tethered anions. The increase in nanoparticle loading improves the connectivity of the cationic surface transport pathways at low nanoparticle concentrations. High nanoparticle loadings were observed to suppress ion mobility because of the strong cation-anion interaction and steric hindrance effects. It was further observed that high dielectric constant solvents facilitate the dissociation of cations, leading to higher ionic conductivity.

Another novel strategy is to develop locally ordered and rigid channels for ion transport with semicrystalline poly-zwitterionic (polyZI) materials, in which the Li^+^ transport number achieves up to 0.67 (Jones et al., 2022). This recent study demonstrated that formation of a nanoscale crystal phase occurs in the polyZI tethered with the zwitterion imidazolium-trifluoromethanesulfonamide (Im-TFSI), which is ascribed to the weak Coulombic interactions that prevent the pendant zwitterions from noncovalent cross-linking. The disparity in size between Li^+^ and the tethered anions promotes sufficient free volume for Li^+^ transport but not for the much larger TFSI¯, resulting in large selectivity for Li^+^ transport. These special confined conditions enable the decoupling of Li^+^ from the slow segmental relaxation in crystalline conductive pathways, although the transport of Li^+^ in the amorphous phase was also observed in these systems. Dating back to earlier studies, the influences of polyZI chemistry (Lind et al., 2016; Taylor et al., 2021) and ionic organization (Keith and Ganesan, 2020) on Li^+^ transport were investigated in theoretical and experimental studies. The experimental results showed improved room temperature ionic conductivities and elastic modulus. This was explained by the enhanced cation/anion dissociation and physical cross-linking within the polymers due to the strong interactions between zwitterions and the mobile ions (Lind et al., 2016; Taylor et al., 2021). Classical MD simulations of polyZIs with distinct organizations indicated that the counterion of the terminal moiety of the zwitterions possesses higher diffusivity than the counterion of the moiety adjacent to the backbone (Keith and Ganesan, 2020). Interestingly, the mobility of the former was found to be correlated with the ion-association behavior, while the latter transported coupling with a cage relaxation. These findings provide crucial insights into this emerging class of ion-containing materials.

In concluding this section, it is important to mention the origin of the difference between charge diffusivity computed from conductivity measurements and that determined by NMR. This is typically quantified with the Haven ratio, $H=D_{i}/D_{\sigma }$ , in which $D_{i}$ is the self-diffusivity measured by NMR without including all the ionic correlation terms, whereas $D_{\sigma }$ the diffusivity derived from impedance spectroscopy using the Nernst-Einstein equation. The interested reader is referred to the excellent perspective (Fong et al., 2021). In ionic liquids that are salts having a melting point below 100°C, an H>1 is explained by the movement of the cation-anion pairs that contribute to ion diffusion, but not to conductivity. However, this explanation was challenged by results from atomistic MD simulations (Kashyap et al., 2011). Further experimental studies of polyILs revealed that the major mechanism suppressing ionic conductivity is due to the strongly correlated movement of mobile ions with a like charge (Popov et al., 2020). Recently, simulations of ILs and polymerized ionic liquids with various degrees of polymerization (N) revealed that the strong distinct correlations between neighboring cations in the backbone of polyILs lead to distinct cation-cation diffusivity that is larger than the self-diffusivity of cations in magnitude and independent of N at larger N (Zhang et al., 2020b). Hence, the inverse Haven ratio shows a maximum at N = 3. Moreover, the authors demonstrated that the ideal transference numbers decrease with increasing N for large N due to the cationic contribution to the conductivity, which challenges the notion that pure polyILs are single-ion conductors. Shen and Hall studied the effects of ion-polymer and ion-ion interactions on the correlation of anion and cation motions by modeling salt-doped homopolymers and block copolymers (Shen and Hall, 2020). The authors found that the increase of ion-monomer interactions gave rise to competing diffusion and ion correlation effects at low salt concentrations and progressively suppressed the conductivity with increasing salt concentration. The stronger ion-ion interactions reduce ion conductivity without being affected by ion concentration (Shen and Hall, 2020). It is worth noting that ion-ion correlations reduce the ionic conductivity in these systems, but enhance the conductivity in superionic ceramics. We would emphasize that one potential strategy is to turn negative ion-ion correlation into positive by tailoring the structure of ion channels and the specific interactions between the ions and lattice. For example, Kumar and coworkers developed one-dimensional ionic channels for anhydrous proton conduction by self-assembly of functionalized organic phosphonic acid and aromatic heterocyclic 1,2,4-triazole molecules (Kumar et al., 2019). The results demonstrate that the one-dimensional conductivity is improved compared with the 3-dimensional channels in Nafion.

## Summary and outlook

Recent significant findings are summarized including the morphology and the ion transport as well as underdeveloped specific interactions within the ion-containing membranes, emphasizing the interplay of morphology and ion transport. We discussed the influences of polymer architectures, tethered ionic groups, the rigidity of the backbone, the solvents, and the additives on the morphology and ion transport of the membranes from a multiscale perspective. Importantly, some novel design strategies were highlighted such as tuning the solvation structures of hydronium/hydroxide ion in hydrated ion exchange membranes, turning negative ion-ion correlation to positive to improve the ionic conductivity in polyILs, etc. In addition, a variety of analytical approaches were reviewed including quantitative cluster analysis, categorization of hopping type, the definitions of dynamic heterogeneity, stringlike cooperative motion, *etc*., for the purpose of gaining mechanistic insight. The remaining challenges include, but not limit to, the design of single-ion conductors, well-defined supramolecular architectures with one-dimensional enhanced ion transport channels, and the understanding of the specific interactions.

To further resolve fundamental issues in theoretically exploring the design rules for advancing the performance of ion-containing materials, we would like to propose several promising directions. First of all, the force field development for atomistic molecular dynamics and coarse-grained models would provide the possibility to gain accurate morphology and dynamical properties from simulations. Specifically, the novel machine learning technique could be extensively applied to the improvement of force fields considering the effects of multi-body interactions, polarizability, and delocalization of charge. New developed force fields for coarse-grained models such as the DFT-based parameterization method (Sepehr and Paddison, 2016), the fragment molecular orbital method (Okuwaki et al., 2018b), the MARTINI (Marrink et al., 2007), and the SPICA (Griffiths and Shinoda, 2019; Miyazaki et al., 2020) could be modified and extended to model ion-containing materials, especially the interfacial phenomena of bipolar membranes. Additionally, machine learning assisted backmapping coarse-grained models to atomistic represented models offers an opportunity to restore details at the atomistic level, leading to an increase in the spatial and temporal scales (Zhang et al., 2019; Li et al., 2020). In view of the temporal limitation of *ab initio* molecular dynamics simulations, it would be potentially promising to develop multi-timestep algorithms to investigate large time scale dynamic properties. To this end, the incorporation of proton transport behaviors into mesoscopic heterogeneity of phase-separated hydrated ionomers is still challenging. It would be promising to introduce various levels of theory into the systems of interest, although the bridging between different levels of theory including coarse-grained approach has yet to be addressed. In addition, an energy decomposition analysis would be a powerful tool for a quantitative interpretation of the specific interactions between fragments/molecules to provide detailed insight into the interactions in terms of chemically meaningful components including: electrostatic, repulsive exchange, induction, and dispersion terms (Zhao et al., 2017).
